# Dynamic transcriptome and histomorphology analysis of developmental traits of hindlimb thigh muscle from *Odorrana tormota* and its adaptability to different life history stages

**DOI:** 10.1186/s12864-021-07677-0

**Published:** 2021-05-20

**Authors:** Yilin Shu, Jun He, Huijuan Zhang, Guangxuan Liu, Shikun Li, Shuaitao Deng, Hailong Wu

**Affiliations:** 1 Collaborative Innovation Center of Recovery and Reconstruction of Degraded Ecosystem in Wanjiang Basin Co-founded by Anhui Province and Ministry of Education, School of Ecology and Environment, Anhui Normal University, Wuhu, China; 2 Provincial Key Laboratory of Biotic Environment and Ecological Safety in Anhui, Wuhu, China

**Keywords:** Development, Adaptability, Hindlimb thigh muscle, Histomorphology, Transcriptome, *Odorrana tormota*, Anura amphibians

## Abstract

**Background:**

Systematic studies on the development and adaptation of hindlimb muscles in anura amphibians are rare. Here, we integrated analysis of transcriptome and histomorphological data for the hindlimb thigh muscle of *Odorrana tormota* (concave-eared torrent frog) at different developmental stages, to uncover the developmental traits of hindlimb thigh muscle from *O. tormota* and its adaptability to different life history stages.

**Results:**

The development of hindlimb thigh muscle from *O. tormota* has the following characteristics. Before metamorphosis, myogenous cells proliferate and differentiate into myotubes, and form 11 muscle groups at G41; Primary myofibers and secondary myofibers appeared during metamorphosis; 11 muscle groups differentiated continuously to form myofibers, accompanied by myofibers hypertrophy after metamorphosis; During the growth process of *O. tormota* from G42 to G46, there were differences between the sexes in the muscle groups that differentiate into muscle fibers, indicating that there was sexual dimorphism in the hindlimb thigh muscles of *O. tormota* at the metamorphosis stages. Some genes and pathways related to growth, development, and movement ability of *O. tormota* at different developmental stages were obtained. In addition, some pathways associated with adaptation to metamorphosis and hibernation also were enriched. Furthermore, integrated analysis of the number of myofibers and transcriptome data suggested that myofibers of specific muscle groups in the hindlimbs may be degraded through lysosome and ubiquitin pathways to transform into energy metabolism and other energy-related substances to meet the physiological needs of hibernation.

**Conclusions:**

These results provide further understanding the hindlimb thigh muscle development pattern of frogs and their adaption to life history stages.

**Supplementary Information:**

The online version contains supplementary material available at 10.1186/s12864-021-07677-0.

## Background

Vertebrate skeletal muscle is a type of large tissue of the body, which is mainly responsible for body movement, energy metabolism, protein storage and protection of internal organs and other important functions [1, 2]. The weight of hindlimb muscles of Anuras accounts for a large proportion of body weight. Compared with the hindlimbs of other vertebrate groups, the hindlimbs of anuras have stronger compliant muscles, which increases the movement ability [3–5]. Anuran’s locomotor performance in walking, jumping, and swimming varies from species to species [6–8]. It is closely related to habitat and morphology [6, 9, 10], and essentially, is related to anurans’ own muscle types and physiology [7, 11]. In addition, the muscles of amphibians also show adaptability to life history. For example, skeletal muscles has been shown to degrade to produce glycogen to meet their hibernation needs [12]. Although myogenesis has been studied in vivo and in vitro in many vertebrates [13], studies on the development of hindlimb muscles, especially those of anura amphibians, are usually limited to a few species [14–18]. The timing and pattern of myogenesis vary among anurans, and those differences have been correlated with lifestyles [19]. Most of these studies were focused on *Xenopus laevis* (African clawed frog) and a few species in family Ranidae, but the specific development patterns of different muscle groups have not been systematically described. In addition, most adult anura amphibians have sexual dimorphism and specific developmental stages such as aquatic to terrestrial, metamorphosis, and hibernation. However, few researchers have investigated gender differences in the development of hindlimb muscles and their adaptation to key life history stages.

The development of high-throughput sequencing technologies has allowed genes and pathways involved in the development processes of many vertebrates to be analyzed at the transcriptome level; for example, in pigs [20, 21], chickens [22], bighead carp [23], and *X. laevis* [24]. Analyzing data for tissue morphology of hindlimb muscles, transcriptome, and life history characteristics together may provide a more systematic and accurate insight into the development traits of hindlimb muscles in amphibians and their adaptability to key life history stages.

In this study, *O. tormota* was selected as the research model. We used the histomorphology, mRNA transcriptome, and life history characteristics information for tadpoles and adults at different developmental periods to analyze whether the differences in the growth and development of hindlimb thigh muscles between male and female *O. tormota* exist in the larval stages; whether the differences at different developmental stages exist in the molecular regulation of hindlimb thigh muscle, and what are the law of adaptability of hindlimb thigh muscles to metamorphosis, hibernation and other life-history stages. The results will provide a foundation for further understanding the hindlimb thigh muscle development pattern of mountain and stream frogs and their adaption to life history stages.

## Results

### Histomorphological features of the hindlimb thigh muscles of *O. tormota* at different developmental stages

**Fig. 1:**
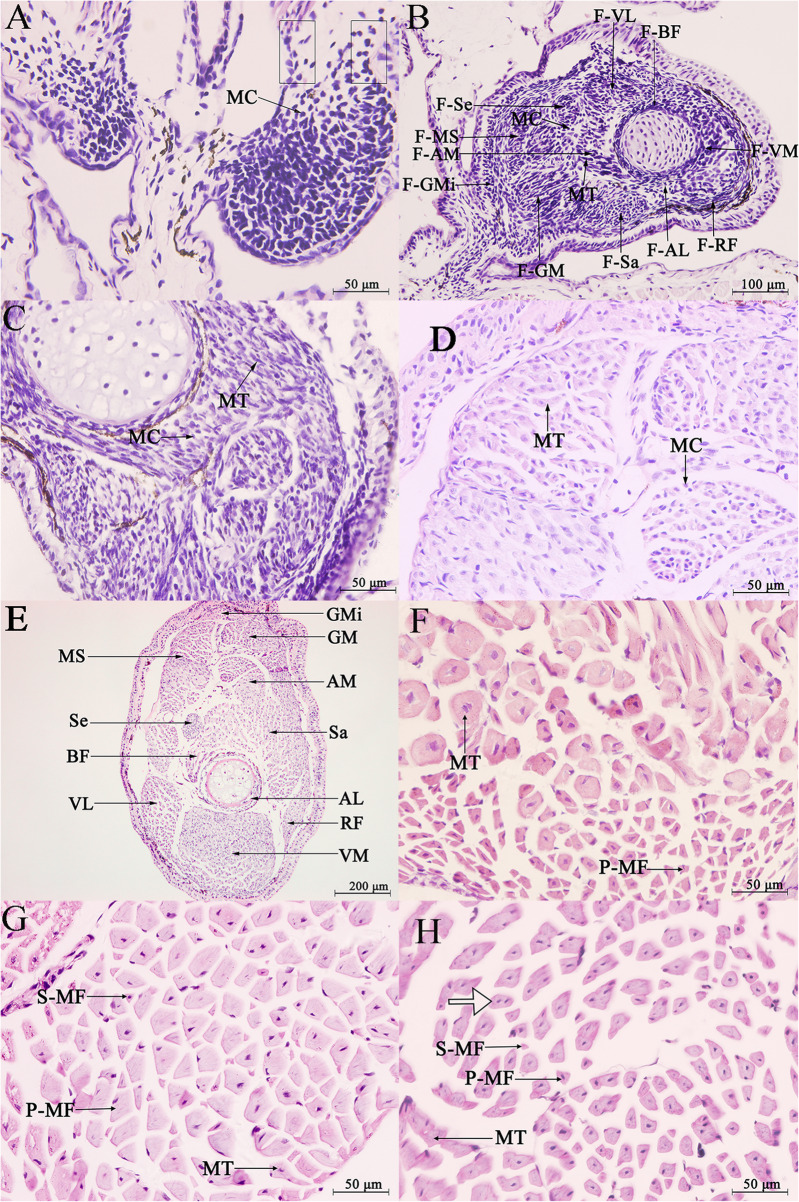
Transverse section of hindlimb thigh muscles in different tadpole stages. Abbreviations: **a** G26; **b** G36; **c** G37; **d** G41; **e** G41; **f** G42; **g** G45; **h** G46. The rectangle represents two streams of myogenous cells. VM, vastus medialis muscle; MS, musculi semimenbranosus; AM, adductor magnus muscle; GM, gracilis major; VL, vastus lateralis muscle; BF, biceps femoris muscle; Sa, sartorius; Se, semitendinosus; GMi, gracilis minor; RF, rectus femoris muscle; AL, adductor longus. MC, myogenous cell; MT, myotube; P-MF, primary myofiber; S-MF, secondary myofiber. F-, corresponding muscle groups in the future

**Fig. 2:**
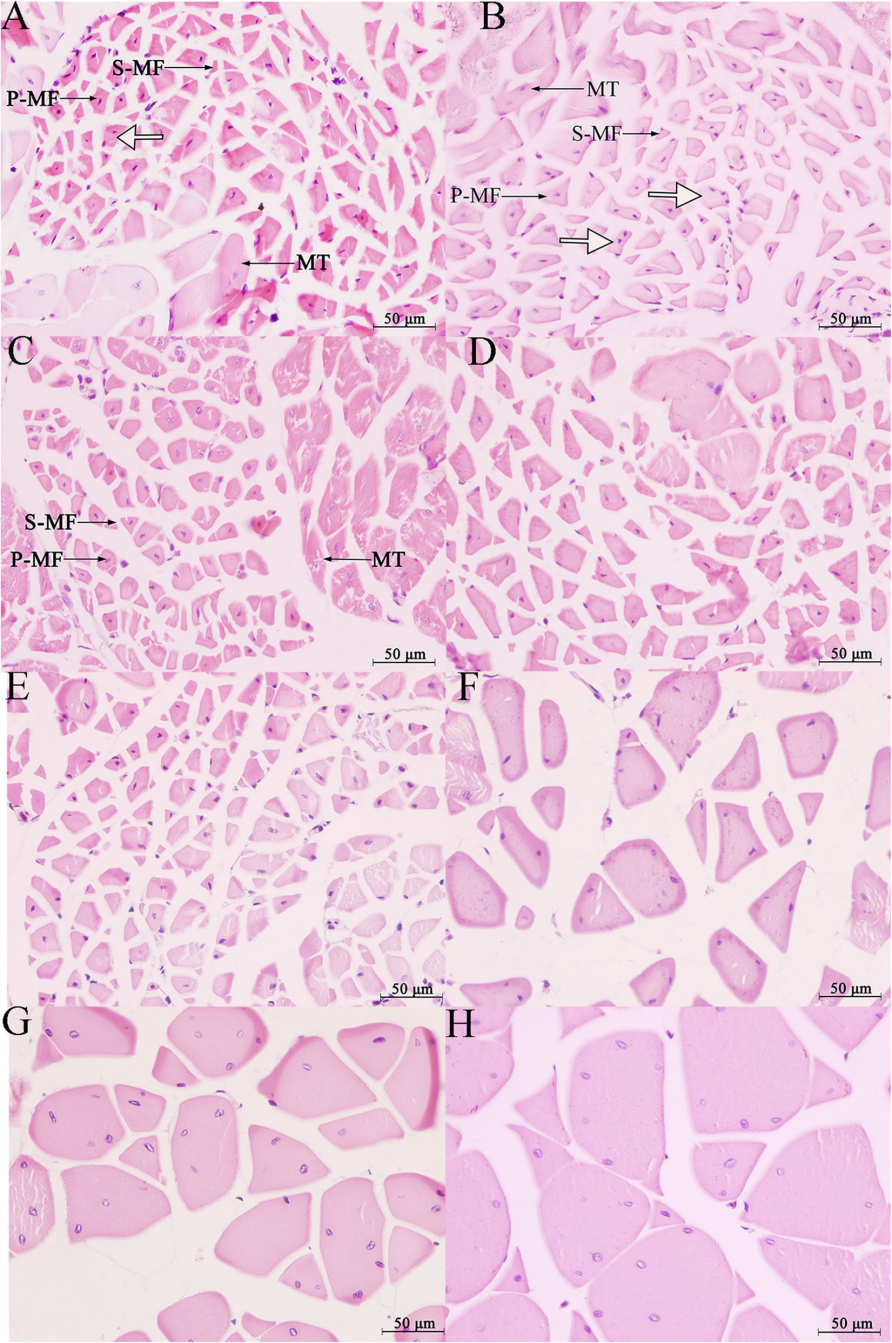
Transverse section of hindlimb thigh muscles. Abbreviations: **a** 1-month old; **b** 3- month-old; **c** 5-month- old; **d** 10-month-old; **e**, 14-month-old; **f** 2-year-old; **g** 3-year-old; **h** 4-year-old. The hollow arrow refers to 2-, 3- or 4-nuclei myofibers. Magnification, 400x (bar = 50 μm)

**Fig. 3:**
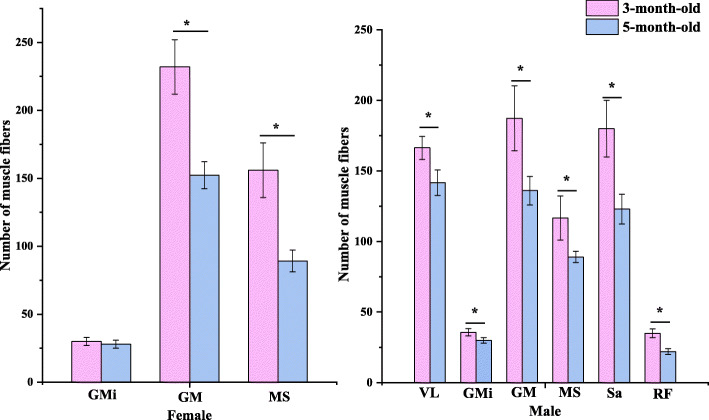
Comparison of the number of muscle fibers at 3-month-old and 5-month-old. MS, musculi semimenbranosus; GM, gracilis major; VL, vastus lateralis muscle; Sa, sartorius; GMi, gracilis minor; RF, rectus femoris muscle. *, *P* < 0.05 (one-way ANNOVA). Error bars indicate the mean ± SEM (standard error of mean)

From G26 to G36, the hindlimb thigh muscles consisted of myogenous cells (Fig. 1a). At G36, 11 groups of pre-myogenic masses of cells and a small number of long cylindrical multinuclear myotubes had formed (Fig. 1b). From G37 to G41, the hindlimb thigh muscles consisted mainly of myotubes (Fig. 1c, d) and the nuclei of multinucleated myotubes moved from the center to the edge until G41 (Fig. 1d). At G41, 11 muscle groups had formed (Fig. 1e). From G42 to 10 months old, the hindlimb thigh muscles consisted mainly of myotubes and myofibers. At G42, six muscle groups in the females (vastus medialis, gracilis minor, gracilis major, adductor magnus, musculus semimembranosus, and sartorius) and four muscle groups in the males (vastus medialis, gracilis minor, gracilis major, and adductor magnus) had differentiated into primary myofibers (Fig. 1f). At G45, a few secondary myofibers had formed around the primary myofibers (Fig. 1g). At G46, the male and female froglets both had a new muscle group that had differentiated into myofibers (biceps femoris for males; vastus lateralis for females) and the fusion of primary and secondary myofibers to form myofibers with two nuclei began to promote myofiber hypertrophy (Fig. 1h). The number of muscle groups with differentiated myofibers in one-month-old froglets after metamorphosis did not change compared with G46. These myofibers had 1 or 2 nuclei (Fig. 2a). Myofibers with three and four nuclei appeared at the 3-month-old stage (Fig. 2b) and these myofibers had increased hypertrophy. Exception for semitendinosus (in the form of tendons) and adductor longus, in the 5-month-old froglets, the other nine muscle groups differentiated into myofibers (Fig. 2c). In the 10-month-old froglets hindlimb thigh muscles, six muscle groups in the females (biceps femoris muscle, gracilis minor, gracilis major, adductor magnus muscle, musculi semimenbranosus and sartorius) and five muscle groups in the males (gracilis minor, gracilis major, adductor magnus muscle, musculi semimenbranosus and sartorius) completely developed into myofibers (Fig. 2d). At 14 months old, all these muscle groups existed as multinucleated myofibers (Fig. 2e–h). From the 14-month to 4-year-old stages, these myofibers underwent further hypertrophy. Comparative analysis of the number of muscle fibers in each muscle group at the 3-month-old (pre-hibernation) and 5-month-old (period of hibernation) stages showed that the number of muscle fibers decreased in three muscle groups in the females (gracilis major, musculus semimembranosus, and gracilis minor) and six muscle groups in the males (vastus lateralis, musculus semimembranosus, gracilis minor, gracilis major, sartorius, and rectus femoris) (Fig. 3). Histomorphological features for other developmental stages were presented in Fig. S1.

### Transcriptome sequencing, de novo assembly, and gene annotation

Samples with obvious differences in histomorphological features and unique life history (metamorphosis and hibernation) were selected to screen for genes and pathways related to development and adaptation to the unique life history. The stages for selected samples were as following, G36 (myogenic cell state), G40 (myotube state), G42 (appearing primary myofibers, early metamorphosis), G45 (appearing secondary myofibers, late metamorphosis), 3-month-old (appearing 3 and 4 nuclear myofibers, pre-overwinter), 5-month-old (9 muscle groups differentiating into myofibers, overwinter), 14-month-old (all these muscle groups completely developing into multinucleated myofibers), and 2-year-old (myofibers undergoing further hypertrophy, adult). The correlation analysis of gene expression levels in the 18 hindlimb thigh muscle samples showed that the correlation of biological duplication was very high (*R* = 0.96–1.00) (Fig. S2). After filtering out the low-quality reads and trimming the adaptor sequences, we obtained 846,522,958 valid reads from the 18 libraries (Table S1). Quality control checks for each library showed that the clean sequences were of high quality, with Q30 values > 92.65% for all libraries. A total of 35,974 genes were identified with lengths of 201–74,670 bp (average length 403 bp) and N50 of 1151 bp. All genes were annotated against the GO, KEGG, Pfam, Swiss-Prot, eggNOG, and NR databases, which returned 9668 (26.87%), 9104 (25.31%), 8884 (24.7%), 9092 (25.27%), 10,582 (29.42%), and 12,388 (34.44%) matches, respectively.

### Time series analysis of gene expression

**Fig. 4:**
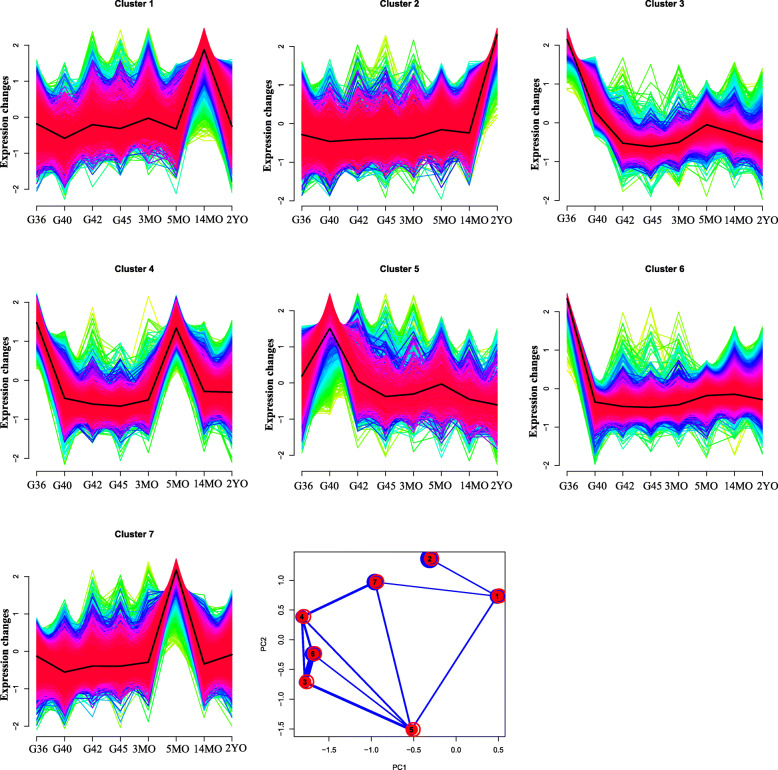
7 clusters obtained by time series analysis and PCA analysis. 3MO, 3- month-old; 5OM, 5-month- old; 14OM, 14-month-old; 2YO, 2-year-old

Samples at eight different developmental stages formed seven time series profiles of gene expressions (Fig. 4). The GO functional enrichment analysis revealed significant differences among the different profiles (Table S2). Profile 2 contained many terms related to muscle growth, development, and movement, including structural constituent of muscle, myofibril assembly, skeletal muscle thin filament assembly, muscle fiber development, locomotory behavior and muscle contraction. The different profiles may provide new molecular characteristics related to muscle development and its adaptation to life history.

### Analysis of differentially expressed genes (DEGs)

A total of 11,482, 8352, 1254, 1911, 6865, 9561, and 8032 DEGs were detected in G36 vs G40, G40 vs G42, G42 vs G45, G45 vs 3- month -old, 3-month-old vs 5-month-old, 5-month-old vs 14-month-old, and 14-month-old vs 2-year-old adult comparisons, respectively (fold change ≥2; adjusted *P* value < 0.05) (Table S3).

**Fig. 5:**
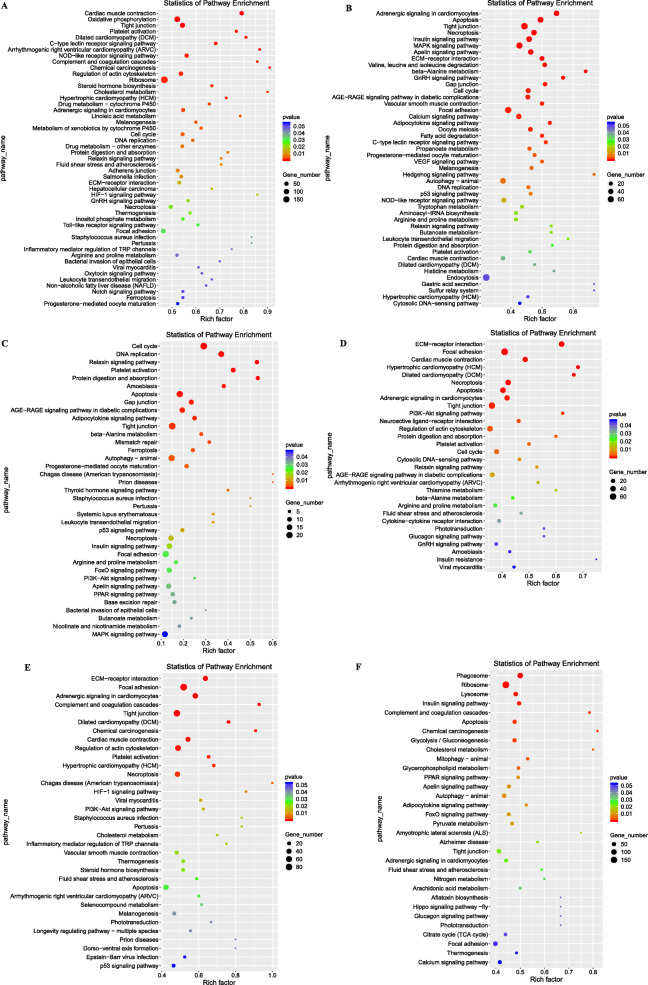
KEGG pathways enriched in six comparable groups. **a** G36 vs G40 group. **b** G40 vs G42 group. **c** G45 vs 3-month-old group. **d** 3-month-old vs 5-month- old group. **e** 5-month- old vs 14-month-old group. **f** 14-month-old vs 2-year-old group

Among the DEGs in the G36 vs G40 comparison, 280 had significantly enriched GO terms (*P* < 0.05), including cell proliferation, nervous system development, immune system process, skeletal muscle fiber development, cell division, hematopoietic progenitor cell differentiation, and chondrocyte differentiation (Table S4), among which 18 terms were related directly to muscle growth and development (Table S5). We screened out 141 DEGs that may be related to muscle growth and exercise, including *MyoD*, *MyoG*, *MYF6*, *KLHL40*, *KLHL41*, *ALDOA*, *OBSCN*, and *TPM3* (Table S6). 11,482 DEGs were significantly enriched in 49 KEGG pathways (*P* < 0.05), including tight junction, focal adhesion, notch signaling pathway, oxytocin signaling pathway, and ECM–receptor interaction. (Fig. 5a).

Among the DEGs in the G40 vs G42 comparison, 181 had significantly enriched GO terms (*P* < 0.05), including skeletal muscle contraction, myosin filament, motor activity, regulation of myoblast differentiation, glycogen metabolic process, and glycogen biosynthetic process (Table S4), among which 21 terms were related directly to muscle growth, development, and exercise (Table S5). We screened out 149 DEGs that may be related to muscle growth and exercise, including *MYF6*, *MEF2D*, *MEF2C*, *MYH8*, *MYH4*, *MYH3*, *MAIA*, *TNNI1*, and *MYOZ3* (Table S6), and 36 DEGs that may be related to glycogen metabolism, glycolysis process, and energy metabolism, including *PGM5*, *PYGB*, *PYGM*, *PRKAG2*, *PRKAG3*, *AMPD1*, and *OXCT1*. Thirty-six of these DEGs were up-regulated in G42 (Table S7). 8352 DEGs were significantly enriched in 46 KEGG pathways (*P* < 0.05), including tight junction, insulin signaling pathway, MAPK signaling pathway, ECM–receptor interaction, focal adhesion, and hedgehog signaling pathway (Fig. 5b).

Among the DEGs in the G42 vs G45 comparison, 178 had significantly enriched GO terms (*P* < 0.05), including myosin filament, cardiac muscle hypertrophy, skeletal muscle thin filament assembly, negative regulation of myoblast differentiation, and cardiac myofibril assembly (Table S4), among which 40 terms were related directly to muscle growth and development (Table S5). We screened out 55 DEGs that may be related to muscle growth and exercise, including *MYH2*, *MYH4*, *LMOD3*, *MYH7B*, *MYH1*, *MYH8*, *MYL4*, and *TTN* (Table S6).

Among the DEGs in the G45 vs 3-month-old comparison, 226 had significantly enriched GO terms (*P* < 0.05), including glucocorticoid receptor binding, glycogen metabolic process, skeletal muscle thin filament assembly, skeletal muscle myosin thick filament assembly, and skeletal muscle fiber development (Table S4), among which 38 terms were directly related to muscle growth and development (Table S5). We screened out 49 DEGs that may be related to muscle growth and exercise, including *MA1A*, *TTN*, *ACTA1*, *MYH4*, *MYLK2*, *PRM*, *SLC8A3*, and *XK70A* (Table S6), and 10 DEGs related to lipid metabolism, namely *CEBPB*, *NOCT*, *PNOLA2*, *PLIN2*, *GM2A*, *PDK4*, *ACACB*, *MLYCD*, *PRKAG2*, and *HPGDS*. Except for *HPGDS*, which was up-regulated at 3-month-old, all the other genes were up-regulated at G45 (Table S8). We also screened out 10 DEGs related to glucose metabolism, namely *PRKAG2*, *PPPLR3C*, *PPPLR3CB*, *CAV3*, *PDK4*, *SLC8B1*, *SLC2A4*, *NCOA5*, *BAD*, and *PCK2*. Except for *NCOA5*, which was down-regulated at 3-month-old, all the other genes were up-regulated at G45 (Table S8). 1911 DEGs were significantly enriched in 37 KEGG pathways (*P* < 0.05), including thyroid hormone signaling pathway, P53 signaling pathway, insulin signaling pathway, focal adhesion, FoxO signaling pathway, PI3K-Akt signaling pathway, MAPK signaling pathway, PPAR signaling pathway, and adipocytokine signaling pathway (Fig. 5c).

Among the DEGs in the 3-month-old vs 5-month-old comparison, 255 had significantly enriched GO terms (*P* < 0.05), including ATP metabolic process, muscle cell development, lysosome, autophagy, ubiquitin ligase complex, glycogen catabolic process, glucose metabolic process, and extrinsic apoptotic signaling pathway (Table S4), among which 47 terms were related to directly muscle growth and development (Table S5). We screened out 144 DEGs that may be related to muscle growth and exercise, including *MYH8*, *MYH4*, *MYH6*, *TWF2-A*, *SCO1*, *FLOT2*, *TMOD4*, *MSTN*, and *PDGFRA* (Table S6). We detected 21 DEGs related to glycogen synthesis and glucose metabolism that were down-regulated at 5-month-old (Table S9) and 76 DEGs related to mitochondria and energy metabolism, 74 of which were up-regulated at 3-month-old; the exceptions were *KDM5C* and *HSD17B14*, which were up-regulated in 5-month-old (Table S9). We also found 90 DEGs related to protein degradation in lysosome and ubiquitination pathways, most of which were up-regulated at 5-month-old; the exceptions were *CTSK*, *CAT-1*, *RNF7*, *ASB15*, *ANAPC16*, and *UBE2C*, which were up-regulated at 3-month-old (Table S9). 6865 DEGs were significantly enriched in 30 KEGG pathways (*P* < 0.05), including ECM–receptor interaction, focal adhesion, apoptosis, tight junction, and PI3K-Akt signaling pathway (Fig. 5d).

Among the DEGs in the 5-month-old vs 14-month-old comparison, 375 had significantly enriched GO terms (*P* < 0.05), including skeletal muscle contraction, skeletal muscle cell differentiation, skeletal muscle thin filament assembly, and positive regulation of myoblast fusion (Table S4), among which 44 terms may be related to muscle growth and development (Table S5). We screened out 215 DEGs that may be related to muscle development and exercise, including *MSTN*, *MYBPC2*, *IGFBP4*, *IGFBPL1*, *MYOG*, *MYF6*, *MYF5*, and *PDLIM3* (Table S6). 9561 DEGs were significantly enriched in 34 KEGG pathways (*P* < 0.05), including ECM–receptor interaction, focal adhesion, tight junction, PI3K-Akt signaling pathway, and p53 signaling pathway (Fig. 5e).

Among the DEGs in the 14-month-old vs 2-year-old adult comparison, 429 had significantly enriched GO terms (*P* < 0.05), including muscle contraction, skeletal muscle myosin thick filament assembly, myofibril assembly, adult walking behavior, and muscle fiber development (Table S4), among which 55 terms were related directly to muscle growth, development, and exercise (Table S5). We screened out 192 DEGs that may be related to muscle development and exercise, including *CAPZA1*, *ACTN4*, *SYNE2*, *SlC4A7*, *MYL12A*, *MEF2D*, *FBXO40*, and *MSTN* (Table S6). 8032 DEGs were significantly enriched in 32 KEGG pathways (*P* < 0.05), including insulin signaling pathway, glycerophospholipid metabolism, PPAR signaling pathway, FoxO signaling pathway, focal adhesion, and hippo signaling pathway-fly (Fig. 5f).

### RT-qPCR verification of randomly selected DEGs

**Fig. 6:**
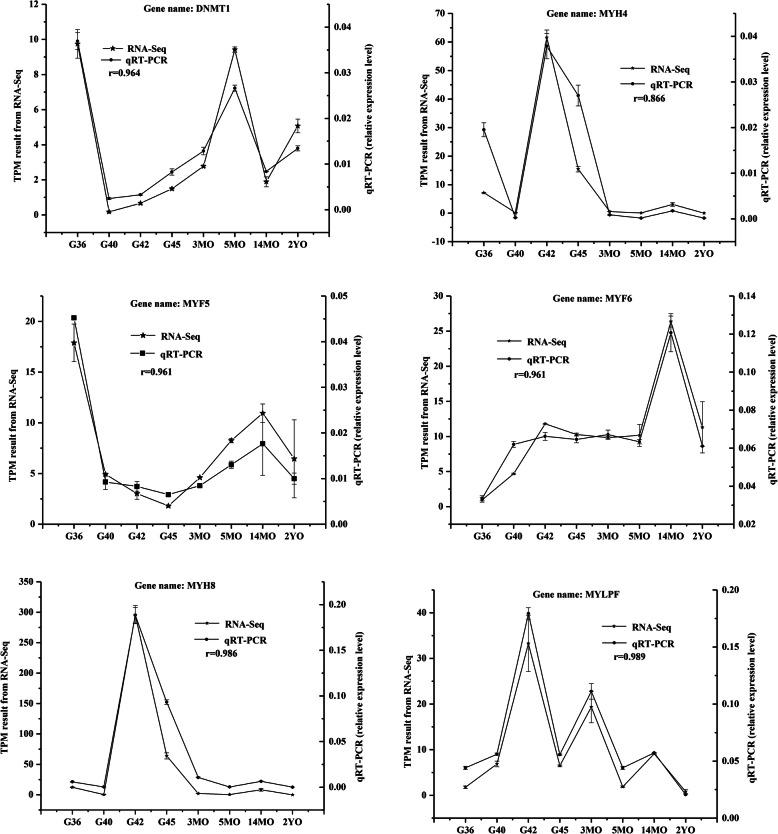
Validation of six DEGs by RT-qPCR. The *r* value represents Pearson’s correlation coefficient between two methods

To confirm the RNA-seq results, we randomly selected five DEGs (*MYH4*, *MYH8*, *MYLPF*, *MYF6*, and *MYF5*) related to muscle development and growth and one DEG (*DNMT1*) related to DNA methylation for verification by RT-qPCR. The RT- qPCR and RNA-seq data showed the selected genes had similar expression trends. Relative expression changes of qRT-PCR data were highly (*r* = 0.87–0.99) correlated with RNA-Seq data (Fig. 6), suggesting the reliability of the RNA-Seq method.

## Discussion

### Developmental histomorphological features of hindlimb thigh muscles of *O. tormota*

The hindlimb thigh muscles of *O. tormota* contained mainly myogenous cells from G26 to G36, myotubes from G37 to G41, and had formed 11 muscle groups at G41. These results are consistent with those reported for tissue sections of the tadpole stages of several anura amphibians [17], indicating that *O. tormota* also grew by muscle cell proliferation and differentiation before metamorphosis [18]. From G42 to 10-month-old, the hindlimb thigh muscles of *O. tormota* contained mainly myotubes and muscle fibers, which is consistent with a previous study that found that the hindlimb muscles of *Rana pipiens* and *X. laevis* grew mainly through hypertrophy and proliferation of muscle fibers [15]. After 14 months, the hindlimb thigh muscles of *O. tormota* contained only muscle fibers, implying that *O. tormota* maintained its muscle fiber hypertrophy after 14 months. During the growth process of *O. tormota* from G42 to G46, there were differences between the sexes in the time when muscle fibers appeared in different muscle groups. There also were differences in the types of muscle groups that existed completely in the form of muscle fibers between 10-month-old male and female froglets. These results indicated that there was sexual dimorphism in the hindlimb thigh muscles of *O. tormota* at the metamorphosis and juvenile frog stages, but the reasons for these differences need to be explored further.

### Enriched genes and pathways related to development and adaptation of life history of *O. tormota*

Enriched GO terms in the G36 vs G40 comparison included nervous system development, immune system process, chondrocyte differentiation, and chondrocyte differentiation, indicating that the immune, bone, nervous, and circulation systems had developed in the hindlimb thigh muscles. The notch signaling pathway has been shown to be involved in somitogenesis, muscle development, and the proliferation and cell fate determination of muscle stem cells during regeneration [25], and the oxytocin signaling pathway was found to play key roles in skeletal muscle regeneration and homeostasis [26]. It also has been reported that oxytocin had important roles in myocardial development [27]. The extracellular matrix (ECM) regulates cell adhesion, differentiation, and proliferation [28], and ECM–receptor interactions have been shown to have a regulatory effect on the growth and development of chicken breast muscles [29]. Tight junctions are barriers that restricted the passage of fluids, proteins, and free ions into and out of paracellular pathways and are necessary for animal growth and development [30]. Focal adhesion, which is the connection between cytoplasm and ECM, plays important roles in cell signal transduction and the stability of cell membrane function [31]. In this study, the hindlimb thigh muscles contained mainly myogenic cells at G36, and mainly myotubes at G40. Among the DEGs, the enriched KEGG pathways included notch signaling pathway, oxytocin signaling pathway, ECM–receptor interaction, tight junction, and focal adhesion. These signaling pathways may play important roles in the differentiation of muscle cells into myotubes and myotube differentiation in the hindlimb thigh muscles of *O. tormota*. In the GO enrichment analysis, 141 DEGs that may be related to muscle growth and movement were screened out. Previous studies have shown that *TCF7L2*, *HES2*, and *HES5* promoted cell proliferation in smooth muscles [32, 33]. *MYOG* also was found to be activated in the early stage of muscle development and acted as a muscle differentiation factor to regulate the differentiation formation stage [34]. *MSTN* and *IGF-I* had feedback regulation to coordinate and control cell proliferation [35, 36]. In G36, the expressions of *TCF7L2*, *HES2*, *HES5*, and *MSTN* were up-regulated, which combined with the proliferation and differentiation characteristics of muscle cells, suggested that these genes may be related to muscle growth and the proliferation and differentiation of muscle cells in the hindlimb thigh muscles of *O. tormota* at this stage. *MYF6* expression has been shown to promote myotube formation [37]. *MyoD* is essential for the differentiation and formation of myogenic precursor cells, and without it, skeletal muscle was not formed [38]. *TTN* was shown to be related to the development of muscle fibers [39], *KLHL40* was associated with myofibril assembly [40], and *OBSCN* was shown to be involved in sarcomere formation [41]. The expressions of *MYF6*, *MyoD*, *TTN*, *KLHL40*, and *OBSCN* were up-regulated in G40. The main morphological characteristics in G40 were myotube fusion and differentiation, suggesting that up-regulated genes related to muscle growth may affect the myotube fusion and differentiation in the hindlimb thigh muscles of *O. tormota* at G40.

In the G40 vs G42 comparison, 149 DEGs that may be related to muscle growth and exercise were screened out. *MYF6* expression was shown to promote myotube formation [37], *MEF2D* promoted muscle tissue development [42], and *FLOT2* was found to be related to myotube fusion [43]. *LBX1* was related to muscle development and was shown to control the migration of myoblasts to the limbs [44]. *MYBPC3* was related to muscle fiber assembly [45]. The expressions of *MYF6*, *MEF2D*, *LBX1*, *FLOT2*, and *MYBPC3* were up-regulated in G42. Hindlimb thigh muscles in G42 consisted mainly of myotubes with a small number of primary muscle fibers appearing in some muscle groups, suggesting that these up-regulated genes may be related to myotube differentiation and formation of primary muscle fibers in the hindlimb muscles of *O. tormota* at this stage*.* The body temperature of a poikilothermal animal changes with changes in the environmental temperature, and their metabolism increases with increases in environmental temperature, within a certain temperature range. We identified 36 DEGs related to glycogen and energy metabolism, and their expressions were up-regulated in G42 (G42: 30 ± 0.08 °C; G20: 20.03 ± 0.12 °C), indicating a promoting effect. Their upregulation may be related to the increased metabolism needed to adapt the increasing environment temperature at G42, and the metabolism of glycogen may maintain energy metabolism under the condition of not entering food during the metamorphosis period. The insulin signaling pathway was related to the efficiency of protein synthesis, and insulin itself also promoted glucose transport [46, 47]. Therefore, the insulin signaling pathway is related to cell differentiation, body growth, and metabolism [48, 49]. Mitogen-activated protein kinase (MAPK) participates in the regulation of cell proliferation, differentiation, and transformation through the phosphorylation of nuclear transcription factors [50]. The hedgehog signaling pathway plays an important role in regulating cell growth and differentiation, tissue and organ formation, and maintenance of homeostasis during embryonic development. This pathway also interacts with the notch signaling pathway and Wnt signaling pathway [51]. Pathways enriched in G40 vs G42 were insulin signaling pathway, MAPK signaling pathway, ECM–receptor interaction, tight junction, focal adhesion, and hedgehog signaling pathway. These pathways may be related to myotube differentiation and formation of primary muscle fibers in the hindlimb thigh muscles of *O. tormota.*

In the G45 vs 3-month-old comparison, 49 DEGs that may be related to muscle growth and movement were screened out. *MA1A*, *ACTA1*, and *TTN* were related to the development of skeletal muscle fibers [52], *MYH1* and *MYH4* were related to myofibers formation [53], and *SYNE1* was related to muscle cell development [54]. There were more muscle fibers and muscle fiber hypertrophy at 3-month-old than at G45, suggesting that the up-regulated genes related to muscle growth also may be related to the formation and hypertrophy of muscle fibers in the hindlimb thigh muscles of *O. tormota* at 3 months old*.* In G45, the tails of *O. tormota* were basically absorbed and no food was taken during the metamorphosis stage, which made *O. tormota* in a hungry-like state. The expressions of nine genes related to glycogen metabolism were up-regulated in G45, further suggesting that the hindlimb thigh muscles of *O. tormota* maintain blood sugar stability by increasing glycogenolysis under conditions of starvation to meet their energy metabolism needs. The thyroid hormone signaling pathway plays an important role in regulating growth, development, and body energy metabolism, and is involved in the metamorphosis development process of amphibians [55]. The *p53* gene is involved in cell differentiation regulation [56, 57]. The FOXO signaling pathway is related to muscle protein degradation and material turnover [58], the PI3K-AKT signaling pathway is related to protein synthesis [59], and the PPAR signaling pathway is related to development, lipid, carbohydrate metabolism, inflammation, and differentiation of many cell types in different tissues [60]. Pathways enriched in G45 vs 3-month-old were thyroid hormone signaling pathway, p53 signaling pathway, insulin signaling pathway, focal adhesion, FOXO signaling pathway, PI3K-AKT signaling pathway, MAPK signaling pathway, and PPAR signaling pathway. These signaling pathways may be related to muscle growth of the hindlimb thigh muscles of *O. tormota* at this developmental stage.

Previous studies have shown that under the condition of low temperature in hibernation, the metabolism of amphibians is reduced to a very low level to adapt to the low temperature and state of starvation [61]. The metabolism reduction in most animals can be achieved mainly by inhibiting the metabolism of skeletal muscle (skeletal muscles make up about 40% of body weight) [61]. The mitochondrial respiration rate was found to decrease during hypoxic hibernation [61]. In the 3-month-old (15 ± 0.29 °C) vs 5-month-old (2.97 ± 0.17 °C) comparison, 21 DEGs related to glycogen synthesis and glucose metabolism were down-regulated at 5-month-old, implying down-regulation of glycogen synthesis and glucose metabolism, and 74 DEGs related to mitochondria and their energy metabolism were down-regulated at 5-month-old, implying down-regulation of mitochondrial energy metabolism. Together, these results suggest that the hindlimb thigh muscles of *O. tormota* also adapt to the hibernation condition of low temperature and state of starvation for 4 months by reducing their energy metabolism. Previous studies had shown that muscle protein degradation was accomplished through four main pathways, the lysosome pathway, calpain pathway, caspase pathway, and ubiquitin proteasome pathway [62–65]. In this study, lysosome and ubiquitinated proteolytic GO terms were enriched. Most of the 90 DEGs related to protein degradation in the lysosome and ubiquitinated pathways were up-regulated at 5-month-old, the except *CTSK*, *CAT1*, *RNF7*, *ASB15*, *ANAPC16*, and *UBE2C*, which were up-regulated at 3-month-old. Among them, the ubiquitination-related genes *UBR4*, *RNF25*, and *Trim63* have been reported to be related to muscle fiber degradation [66–68]. The analysis of histomorphological characteristics showed that the muscle fibers of six muscle groups were significantly reduced in males at 5-months-old compared with males at 3-months-old. In a recent study, skeletal muscle was found to degrade to produce glycogen for amphibians to adapt to hibernation [12]. Therefore, muscle fibers in specific muscle groups of the hindlimb thighs of *O. tormota* may degrade through the lysosome and ubiquitination pathways. The degraded muscle fibers could be converted into energy metabolism and other energy-related substances to adapt to hibernation.

The hindlimb thigh muscles of *O. tormota* at 14-month-old were in the form of muscle fibers. These muscle fibers showed hypertrophy at 14 months compared with their condition at 5 months. At this stage, the sub-adult *O. tormota* could jump flexibly and predation. In the 5-month-old vs 14-month-old comparison, 215 DEGs related to muscle growth and movement were screened out. These genes may be related to muscle fiber development and movement ability of the hindlimb thigh muscles of *O. tormota*. The histomorphological characteristics showed that the muscle fibers had further hypertrophy from 14-month-old to 2-year-old adults. At 2 years, adult *O. tormota* preyed and competed for mating rights in the complex habitat around streams. In the 14-month-old vs 2-year-old comparison, 192 DEGs related to muscle growth and movement were screened out. These genes may be related to the maturation and regeneration of muscle fibers and exercise capacity of the hindlimb thigh muscles of *O. tormota*. Previous studies have shown that the hippo signaling pathway was connected with the TGF-β signaling pathway and could control the size of organs by regulating ubiquitin-mediated proteolysis, cell proliferation, and apoptosis [69]. Therefore, the enriched pathways, insulin signaling pathway, PPAR signaling pathway, FOXO signaling pathway, focal adhesion, and hippo signaling pathway, may be related to the maturation and regeneration of muscle fibers and exercise capacity of the hindlimb thigh muscles of *O. tormota*.

Although only two biological replicates were used for the transcriptome samples in this study, the same feeding conditions, uniform genetic background from having the same parents, distinguished gender, consistent sampling of tissues, and high correlation of the biological replicates (the gene expression levels between samples; *R* = 0.96–1.00) ensured the reliability of the data analysis. The RT- qPCR verification of gene expression also supported the good correlation of sample biological repetition.

## Conclusions

In this study, by integrating tissue morphological characteristics, life history characteristics, and mRNA expression profiles at different developmental stages, we successfully screened genes and pathways related to the growth, development, movement, and adaptation to life history of hindlimb thigh muscles of *O. tormota*. Our results will help to further understand the development patterns of hindlimb thigh muscle in frogs and the molecular mechanisms involved in their adaptation to life history.

## Methods

*Odorrana tormota* (concave-eared torrent frog) is a small sized frog with typical sexual dimorphism. The weight and length of adult females are larger than those of adult males. After metamorphosis, they live mainly on land around streams in mountainous areas [70–74]. Their habitat, life history, breeding methods, and genetic background are relatively well known. Therefore, *O. tormota* is an ideal model for the study of sexual differences in muscle development of Ranidae species and their adaption to life history stages.

### Breeding methods

In the wild mountain stream, a litter of fertilized egg samples containing about 600 eggs was selected and enclosed them with a 40-mesh gauze within about 1 m^2^ for incubation. After the fertilized eggs hatch, these tadpoles were averagely put into 6 breeding ponds (Length*width*height: 0.75 cm*0.45 cm*0.40 cm) constructed near the mountain stream. Each tadpole breeding pond simulated the stream environment, introduced the stream water, kept 6-8 cm of the water depth, and retained the water flowing. The tadpoles raised with egg yolks and boiled vegetable leaves (1:1) twice a day, and each feeding amount was about 10% of the tadpole’s body weight. Tadpoles at the G42 were moved from the tadpole breeding pond to the froglet breeding pond. The tadpoles (G42-G46) stopped eating during the metamorphosis stage. Pebbles were placed on the bottom of the froglet breeding pond (Length*width*height: 2 m*2 m*0.8 m) with a certain slope. Then, the stream water was introduced into the pond with a depth about 5 cm, and the water area and land area ratio was about 2:1. The pool was sealed with a gauze net to prevent the escape of froglets and their edible insects. At the corner of the froglet pond, rocks of different sizes piled up to form gaps to provide hibernating nests for froglets. Finally, the corner was covered with a layer of sand and dead leaves for insulation. The feeding conditions of froglets after hibernation were basically the same as those before hibernation.

### Sample collection

Individuals were raised in the natural environment of the wild habitat from the fertilized eggs stage to the 14-month-old sub-adult stage. A litter of fertilized egg samples containing about 600 eggs was selected for breeding. Tadpoles and frogs were euthanized by MS-222 overdose, and dissected with scissors soaked in a 0.1% diethylpyrocarbonate solution. In order to facilitate research, we divide the development of frogs into three kinds of stages, tadpole stages, froglet stages, and adult stages. According to the criteria for dividing periods defined by Gosner (1960), 46 stages (G1–G46) from fertilization to completion of tadpole metamorphosis were recognized. The age classification criteria for the froglets were as follows: one-month-old, 1 month after metamorphosis; 2-months-old, 2 months after metamorphosis; and so on. *O. tormota* began to appear the hindlimb buds at G26. In this study, G26 to G41 were the pre-metamorphosis stages, and G42 to G46 were the metamorphosis stage. The sex of each sample at the tadpole and the juvenile frog stages was distinguished according to the characteristics of the gonadal tissues [75]. The histological characteristics of the gonads at the different development stages showed that sex differentiation of *O. tormota* began at G34 (Fig. S3). We obtained a total of 220 samples from G26 to 14-month-old froglets (26 developmental stages). There were 5 samples at each stage of sex undifferentiated (G26 to G33), and 5 males and 5 females at each stage after sex differentiation (G34 to 14-month-old). Adult frogs were captured in the stream area near the breeding site. The ages of the adults were judged by skeletochronology with one growth arrest line defined as 2 years old [76] (Fig. S4). Three male and three female frogs at 2 years old, 3 years old, and 4 years old (a total of 18 frogs) were selected as samples. The temperature of the aquatic habitat for the groups before the metamorphosis was measured by placing a thermometer probe 10 cm below the water, and that of the terrestrial habitat for the groups for and after the metamorphosis was measured 10 cm above the ground with the temperature probe to measure the air temperature. The temperatures were measured at 10 am. The temperature of the collected samples is kept similar with the ambient temperature of the habitat by temperature control equipment (KFR-35GW/NhGe3B, Zhuhai Gree Electric Co., Ltd., Zhuhai, China). All samples information is presented in Table S10.

### Analysis of histomorphological features of hindlimb thigh muscles

Three normal-growth tadpoles were selected randomly at each developmental stage before sex differentiation. After sex differentiation, three normal-sized male and three normal-sized female frogs were chosen randomly at each developmental stage. The tissue sections and hematoxylin and eosin (H&E) staining methods were according to Manzano et al. [17]. For each sample, the slices that had the largest cross-sectional area were selected for tissue morphological analysis. The terminology and classification of the muscles was according to Chen et al. and Manzano et al. [17, 77]. An Olympus BX61 microscope (Olympus, Tokyo, Japan) was used to take pictures of the cross-sections of the hindlimb thigh muscles and each muscle group to analyze the tissue morphological characteristics. The number of muscle fibers of each muscle group was counted by Image-Pro Plus 6.0 (Media Cybernetics Inc., Rockville, MD, USA). One-way ANNOVA was used to compare the number of muscle fibers.

### RNA extraction and mRNA sequencing library construction

We selected 18 individuals as samples as follows: two male tadpoles at G36 (myogenic cell state), G40 (myotube state), G42 (early metamorphosis), and G45 (late metamorphosis); two male froglets at 3 months old (pre-overwintering) and 5 months old (overwintering); two male and two female sub-adult frogs at 14 months old; and two male adult frogs at 2 years old. From the tadpole stages to 14-month-old froglets, the muscles from the same part of the hindlimb thighs were selected from both legs and mixed. For the 2-year-old adult frogs, the hindlimb muscles were taken from one leg at the same part as was used for the froglets. Two biological replicates were selected for each developmental stage. Detailed sample information is presented in Table S11. The mRNA sequencing (RNA-seq) data of the OT1MMY2 and OT2MMY2 samples for the 2-year-old male adults were derived from our previously published RNA-seq data OT1MM (SRR6896144) and OT2MM (SRR6896143) [73]. For the other 16 samples, total RNA was extracted using a miRNeasy Mini Kit (Qiagen, Hilden, Germany) following the manufacturer’s procedure. The 16 total RNA samples were treated with RNase-Free DNase I (Qiagen) to remove genomic DNA. Total RNA quantity was analyzed with an Agilent 2100 Bioanalyzer (Agilent Technologies, USA) based on a 28S:18S ratio > 1.0 and RNA integrity number > 8. We constructed 16 cDNA libraries using a TruSeq® RNA Sample Preparation Kit (Illumina, San Diego, CA, USA). The average insert size for the paired-end libraries was 300 bp (± 50 bp). Paired-end sequencing was performed on an Illumina HiSeq 4000 system at LC Sciences (Hangzhou, China).

### Transcriptome data analysis

Cutadapt [78] and Perl scripts were used to discard adapter-contaminated reads, low-quality reads, and reads with undefined bases. De novo transcriptome assembly was performed using Trinity 2.4.0 [79], which assembles transcripts into clusters based on their common sequence contents. The longest transcript in each cluster was chosen as the gene sequence (also called unigene). All the assembled gene sequences were annotated against six databases, Swiss-Prot (http://www.expasy.ch/sprot/), the non-redundant protein sequence database (NR) (http://www.ncbi.nlm.nih.gov/), Gene Ontology (GO) (http://www.geneontology.org), Pfam protein families (http://pfam.xfam.org/), Kyoto Encyclopedia of Genes and Genomes (KEGG) (http://www.genome.jp/kegg/), and eggNOG (http://eggnogdb.embl.de/), using DIAMOND [80] with E-value < 0.00001. Transcripts per kilobase million (TPM) was used to normalize the expression levels of the genes [81]. DEGs were selected with log2 (fold change) > 1 or < − 1 and adjusted *P* value ≤0.05 using edgeR in the R package [82]. GO and KEGG enrichment analyses of the DEGs were performed using in-house Perl scripts. To identify genes with similar functions, time series analysis was performed using Mfuzz v2.34.0 [83] to assign genes to multiple clusters based on similar expression profiles. Genes with high confidence in each cluster were obtained by filtering (membership value ≥0.5), then the GO enrichment analysis was performed.

### Validation of DEGs by qPCR

Six DEGs were selected for verification by RT- qPCR. *RPL32* (ribosomal protein L32) was used as the internal reference. The primer sequences are listed in Table S12 and each sample had 3 replicates. Reverse transcription and amplification were performed using TransScript All-in-One cDNA SuperMix for RT-qPCR (TransGen, China) and GoTaq qPCR Master Mix (Promega, USA) according to the manufacturers’ instructions. The 2^−△△CT^ method was used to calculate the relative expression of the genes. Data were analyzed by Student’s *t*-test and presented as the mean ± SEM of two independent samples.

## Supplementary Information

**Additional file 1: Fig. S1**. Transverse section of hindlimb thigh muscles in tadpole stages

**Additional file 2: Fig. S2**. Pearson correlation of gene expression for all samples

**Additional file 3: Fig. S3**. Hematoxylin-stained cross-sections of the gonads. Note: M, mesonephros; PGC, primordial germ cell; T, testis; O, ovary; Po, primary oocyte; Oc, Ovarian cavity; Sg, Spermatogonia; S, somatic cell; Sv, seminal vesicles; Pc, Primary cavity; Sc, Secondary cavity; Oo, oogonium; MO, month-old; ♀, female; ♂, male

**Additional file 4: Fig. S4**. Hematoxylin-stained cross-sections of the thigh-bone. The black solid arrow refers to the stagnant growth line. Outer edge of bone (OEB). A, 2-year-old; B, 3-year-old; C, 4-year-old. RL and FL represent resorption lines (the division line between endosteal and periosteal zones) and false line, respectively. Magnification, 200x (bar = 100 μm)

**Additional file 5: Table S1**. Summary of generated transcriptome data

**Additional file 6: Table S2**. GO enrichment of 7 time series clusters

**Additional file 7: Table S3**. DEGs in seven comparable groups

**Additional file 8: Table S4**. GO enrichment in seven comparable groups

**Additional file 9: Table S5**. GO terms were related to muscle growth and development in the seven comparable groups

**Additional file 10: Table S6**. DEGs related to muscle growth and exercise in seven comparable groups

**Additional file 11: Table S7**. DEGs related to glycogen metabolism, glycolysis process and energy metabolism at the G40 vs G42 group

**Additional file 12: Table S8**. DGEs related to glucose and lipid metabolism at the G45 vs 3-month-old group

**Additional file 13: Table S9**. DGEs related to glucose metabolic, mitochondria, energy metabolism, lysosome and ubiquitination pathway at the 3-month-old vs 5-month-old group

**Additional file 14: Table S10**. Samples collecting information

**Additional file 15: Table S11**. Samples collecting information for transcriptome analysis

**Additional file 16: Table S12**. qRT-PCR primers for mRNAs

## Data Availability

The raw transcriptome data have been submitted to the NCBI. Sequence Read Archive under accession SRP234059. Transcriptional assembly deposited at GenBank under accession GICS00000000.

## Abbreviations

RT-qRCR: Reverse Transcription Real-time Quantitative PCR
DEGs: Differentially expressed genes
RNA-seq: RNA sequencing
TPM: Transcripts per kilobase million
KEGG: Kyoto Encyclopedia of Genes and Genomes
GO: Gene Ontology
H&E: Hematoxylin and eosin
TGF-β: Transforming growth factor-β
PPAR: Peroxisome proliferators-activated receptors
FOXO: Forkhead box O
MAPK: Mitogen-activated protein kinase
