# Supplementary material for: Dynamic transcriptome and histomorphology analysis of developmental traits of hindlimb thigh muscle from Odorrana tormota and its adaptability to different life history stages
Source: BMC Genomics. 2021 May 20;22:369. doi: 10.1186/s12864-021-07677-0 (PMC8138932; doi:10.1186/s12864-021-07677-0)
Supplement: Supplementary file 1 — Additional file 1: Fig. S1. Transverse section of hindlimb thigh muscles in tadpole stages [file 12864_2021_7677_MOESM1_ESM.pdf]

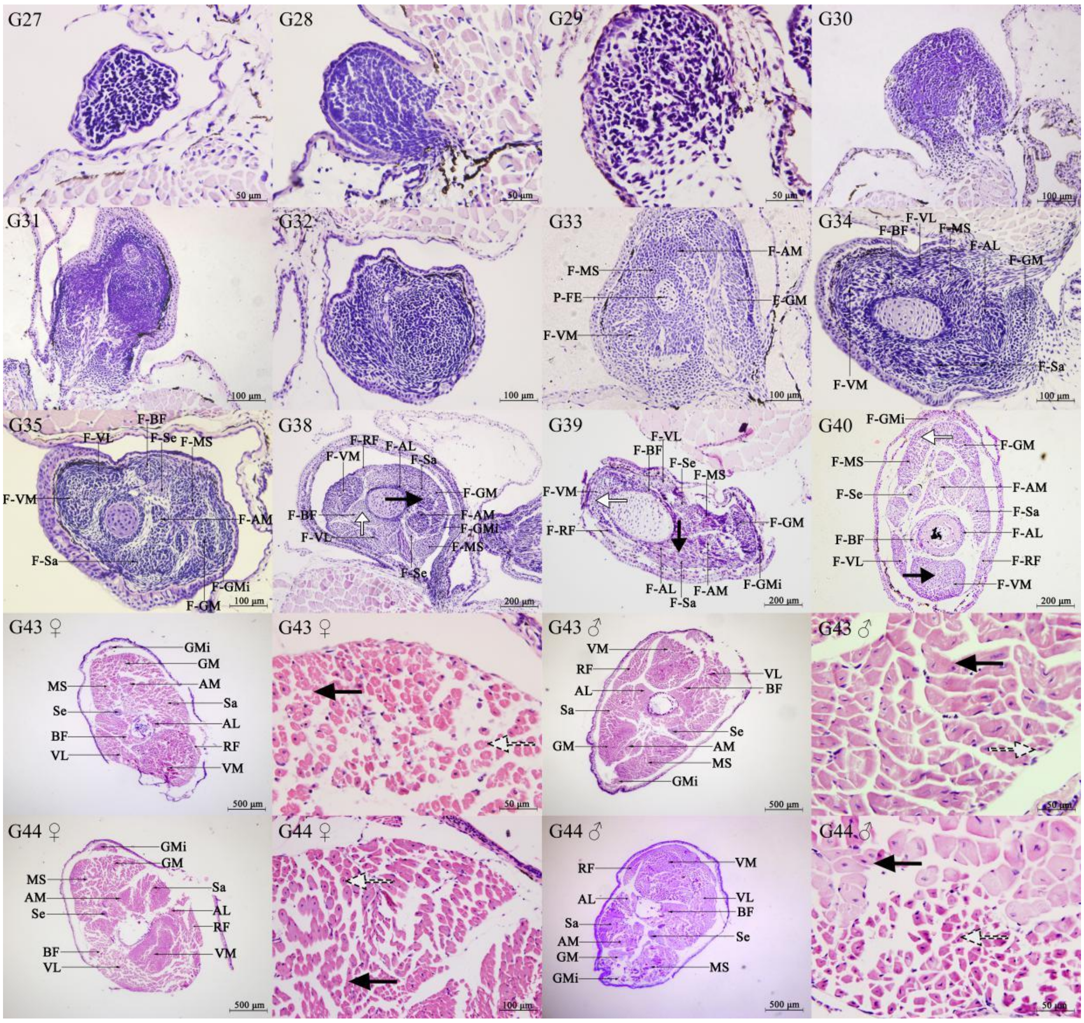

Fig. S1 Transverse section of hindlimb thigh muscles in tadpole stages. P-Fe, presumptive femur; VM, vastusmedialis muscle; MS, musculi semimenbranosus; AM, adductor magnus muscle; GM, gracilis major; VL, vastus lateralis muscle; BF, biceps femoris muscle; Sa, sartorius; Se, semitendinosus; GMi, gracilis minor; RF, rectus femoris muscle; AL, adductor longus. F-, corresponding muscle groups in the future. ♀, female; ♂, male. The black hollow arrow refers to myogenous cells, the solid arrow refers to the myotube, the dashed hollow arrow refers to the primary myofiber.

The histomorphological description of hindlimb thigh muscles at different developmental stages: From G27 to G35, the hindlimb thigh muscles consisted of myogenous cells, which were the proliferation period of myogenous cells. At G33, 4 groups (F-VM, F-MS, F-AM and F-GM) of pre- myogenic masses of cells had formed. At G34, 7 groups (F-VM, F-MS, F-AM, F-GM, F-VL, F-BF and F-Sa) of pre- myogenic masses of cells had formed. At G35, 9 groups (F-VM, F-MS, F-AM, F-GM, F-VL, F-BF, F-Sa, F-Se and F-GMi) of pre- myogenic masses of cells had formed. From G38 to G40, the hindlimb thigh muscles consisted mainly of multinucleated myotubes. The multinucleated myotubes formed by the fusion of myogenous cells in 11 groups of pre-myogenic masses gradually increase. The multinucleated myotubes formed by the fusion of myogenous cells in 11 groups of pre-myogenic masses gradually increase. In G43 and G44, the hindlimb thigh muscles consisted mainly of multinucleated myotubes and myofibers. Six muscle groups in the females (VM, GMi, GM, AM, MS and Sa) and eight muscle groups in the males (VM, GMi, GM, AM, MS, Sa, RF and VL) had differentiated into primary myofibers. The

number of primary myofibers differentiated in each muscle group increased.
