# Supplementary figures and images for: Dynamic transcriptome and histomorphology analysis of developmental traits of hindlimb thigh muscle from Odorrana tormota and its adaptability to different life history stages
Source: BMC Genomics. 2021 May 20;22:369. doi: 10.1186/s12864-021-07677-0 (PMC8138932; doi:10.1186/s12864-021-07677-0)

Pearson correlation between samples

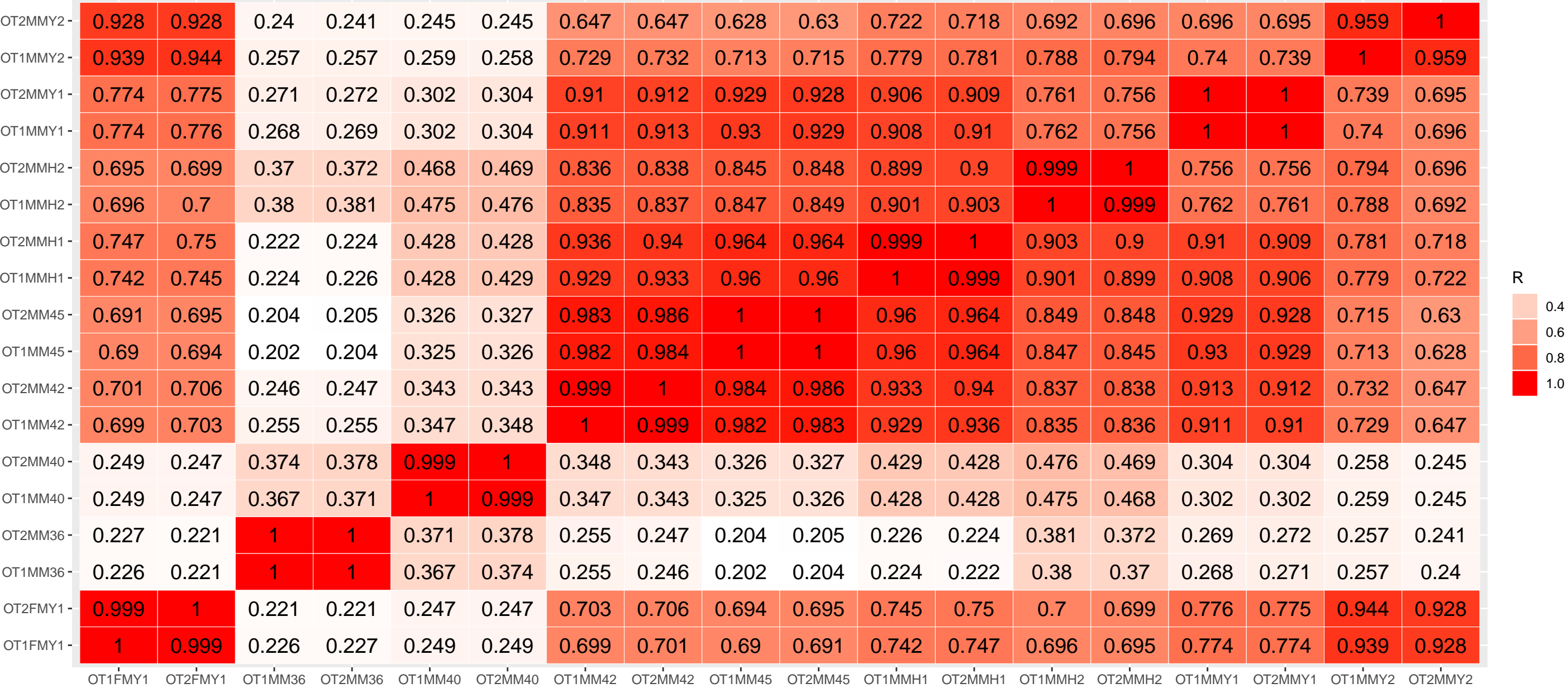

Supplement: Supplementary file 2 — Additional file 2: Fig. S2. Pearson correlation of gene expression for all samples [file 12864_2021_7677_MOESM2_ESM.pdf]

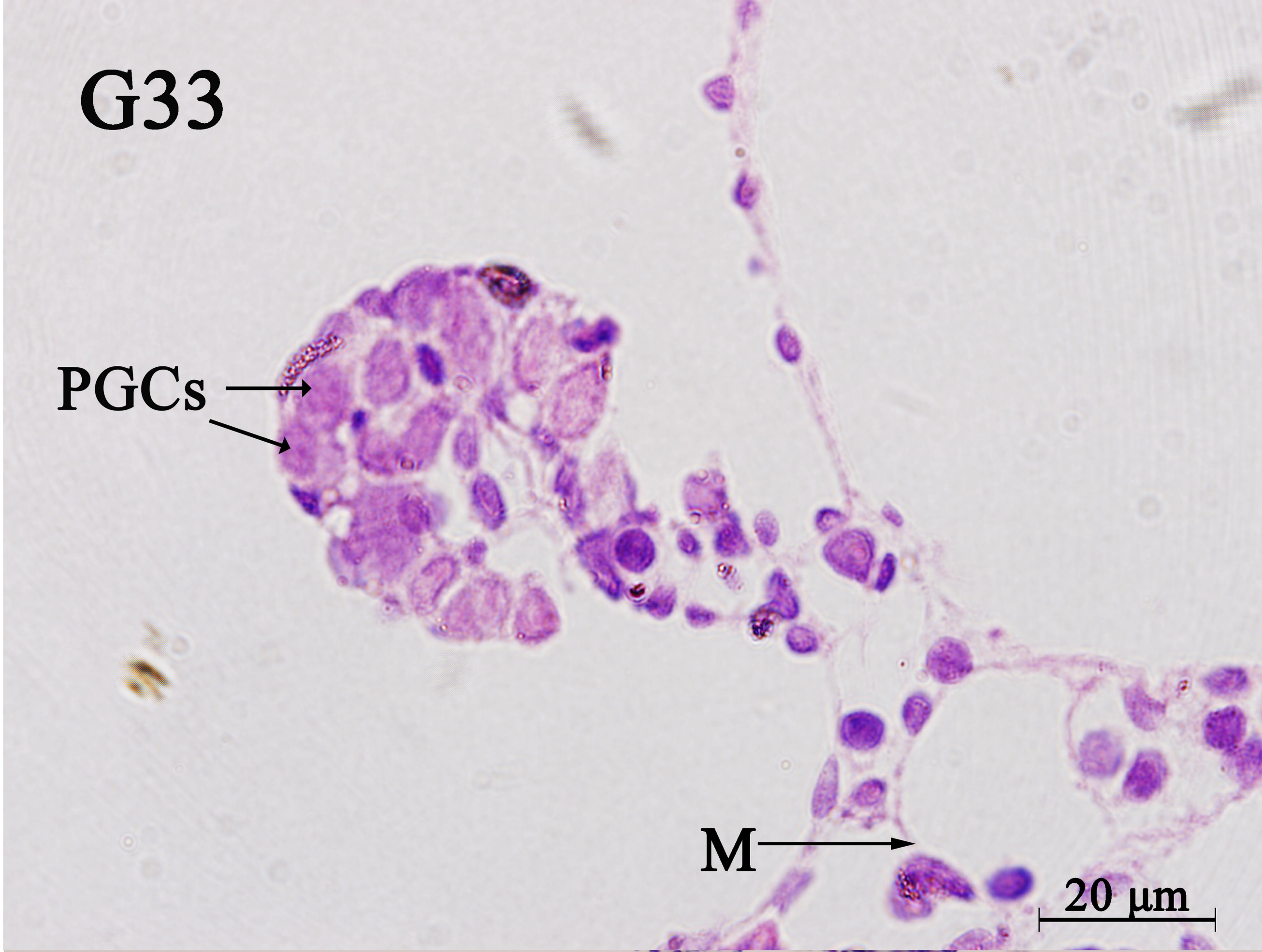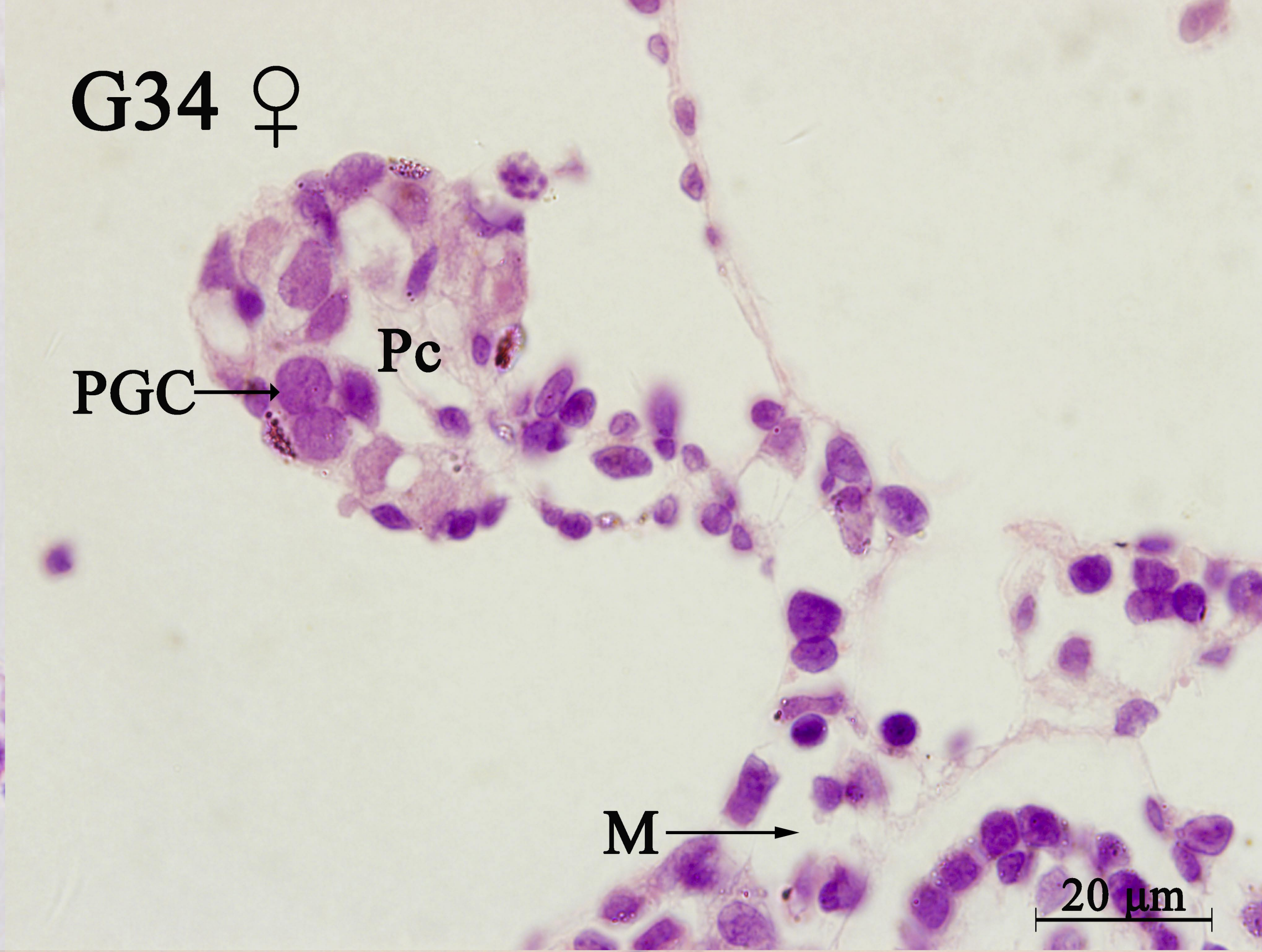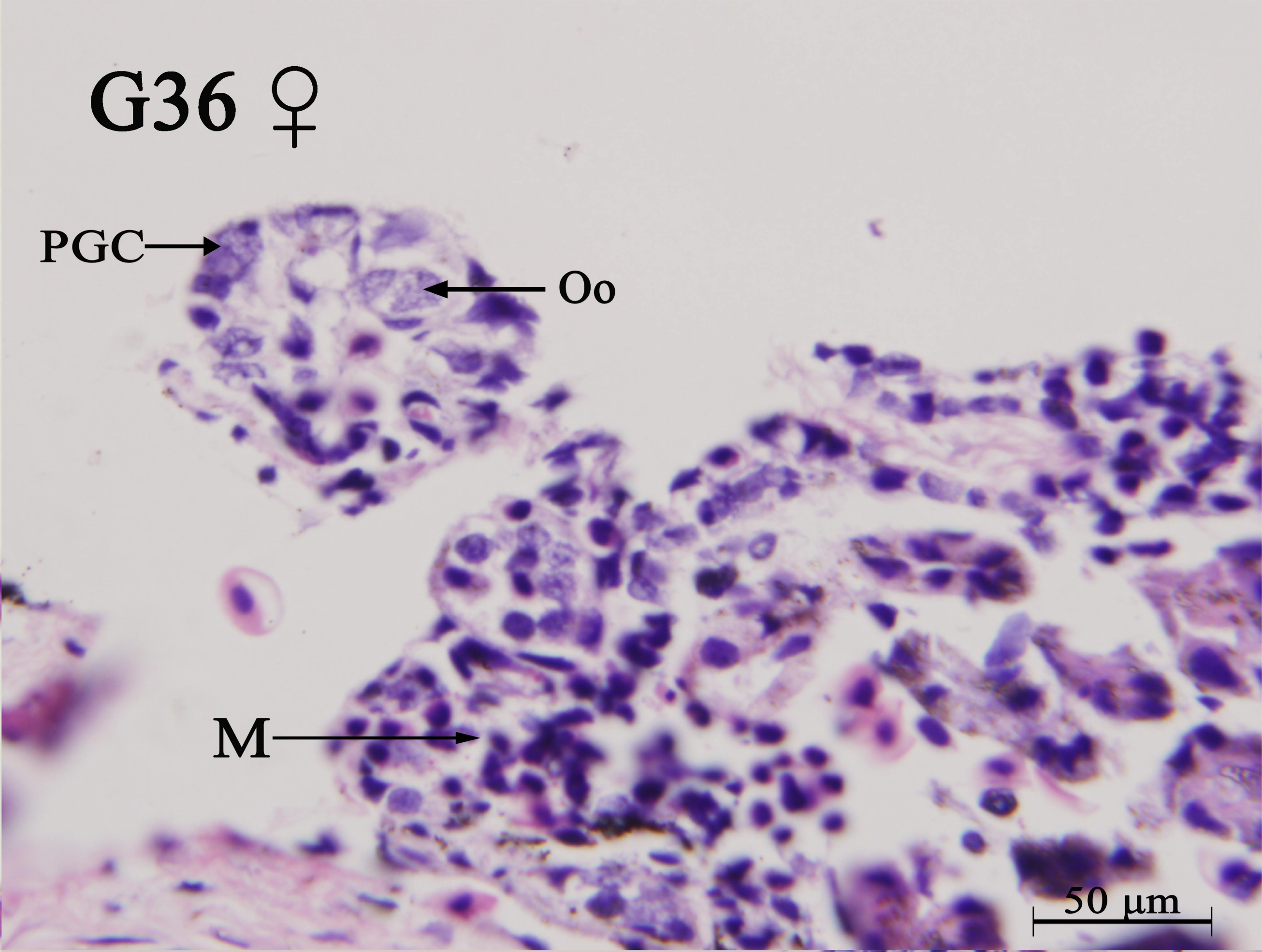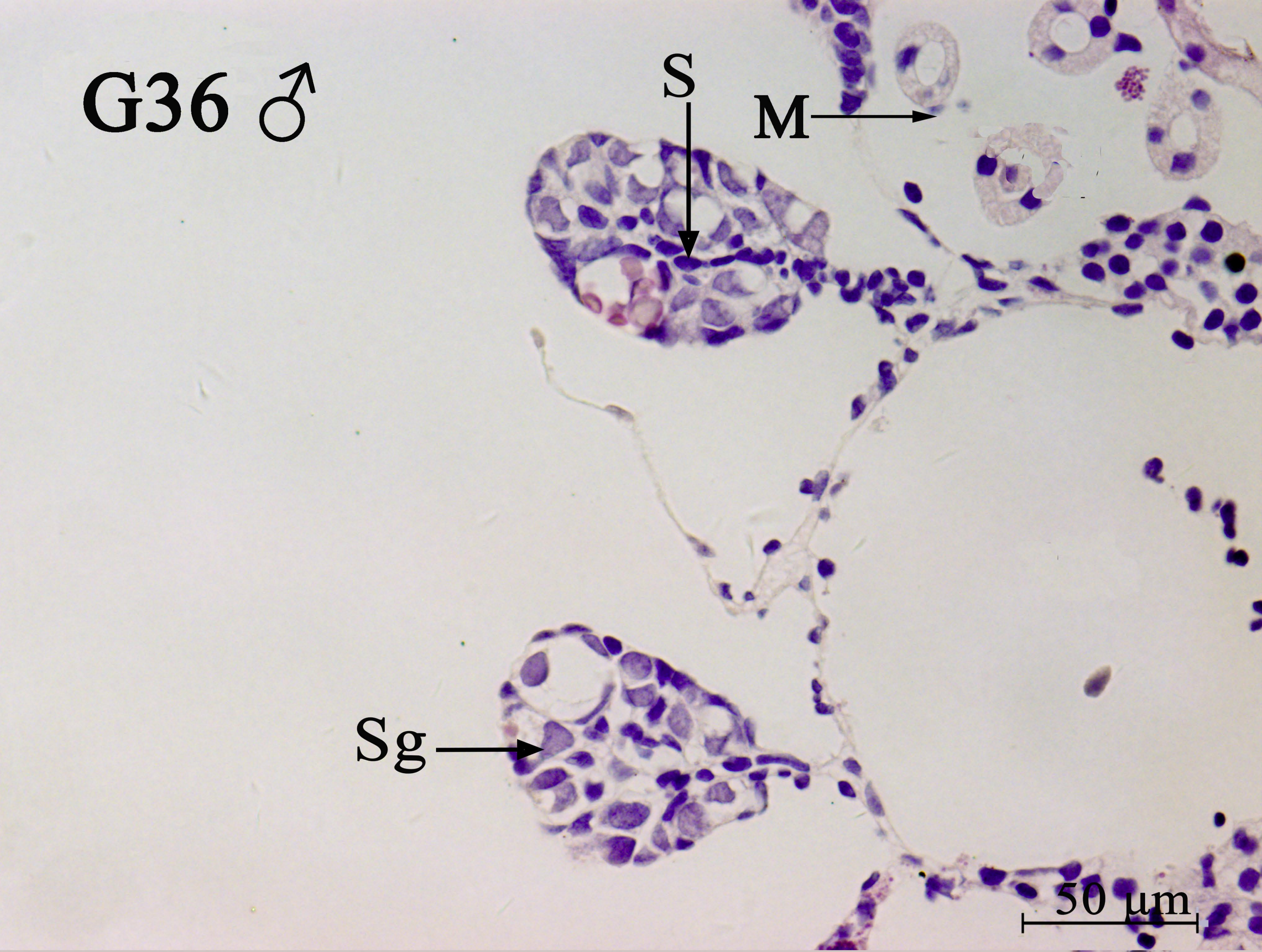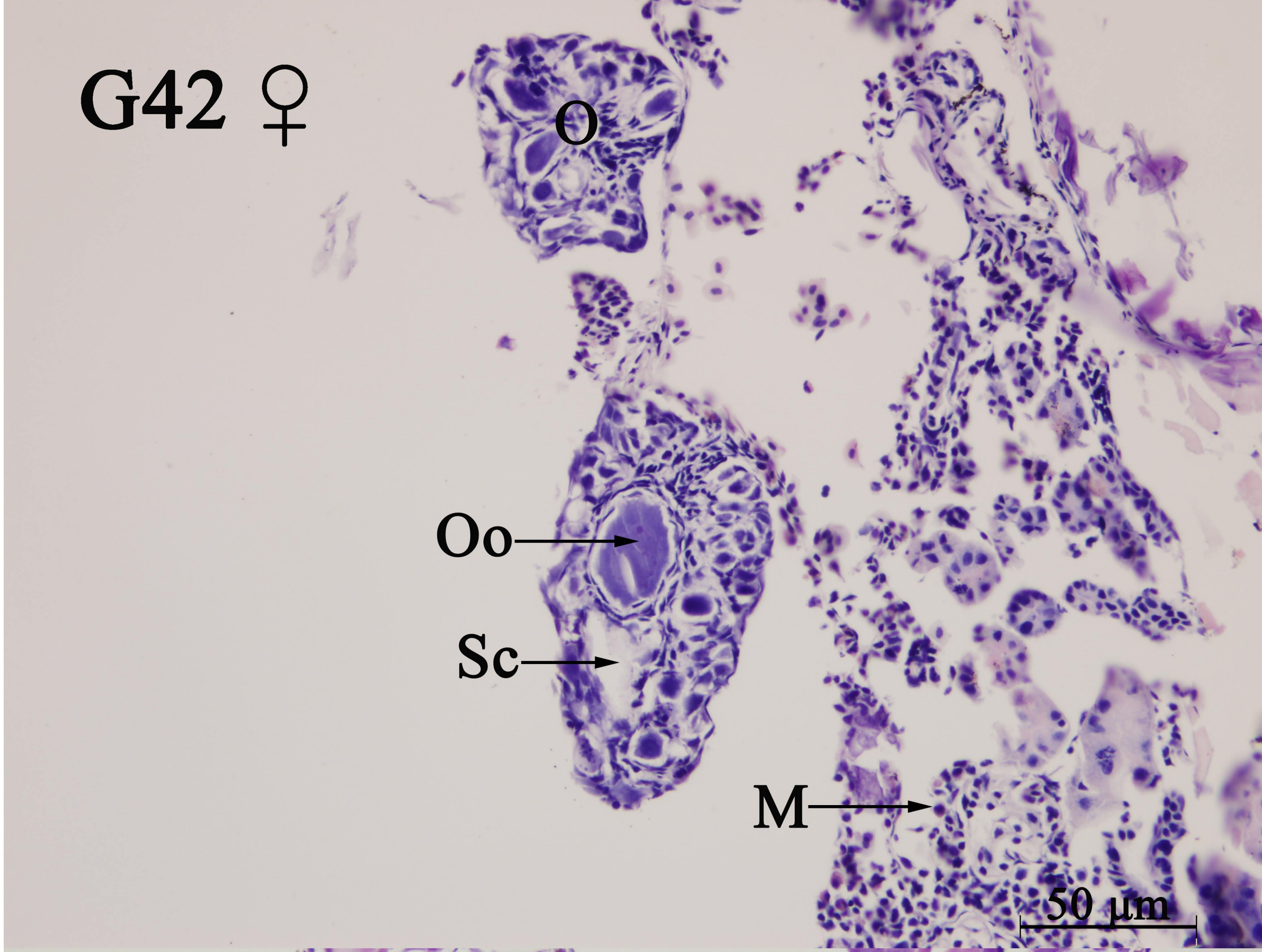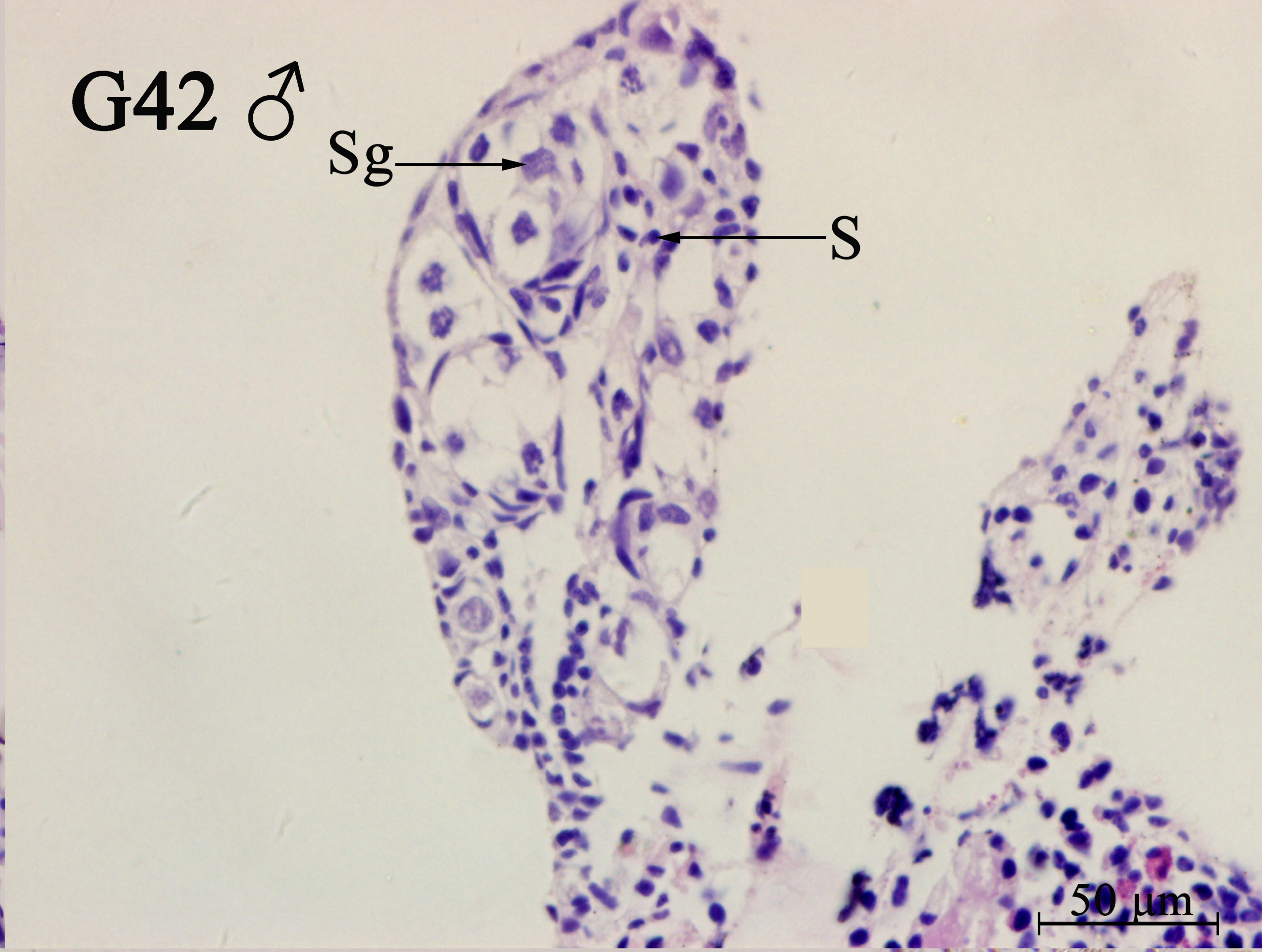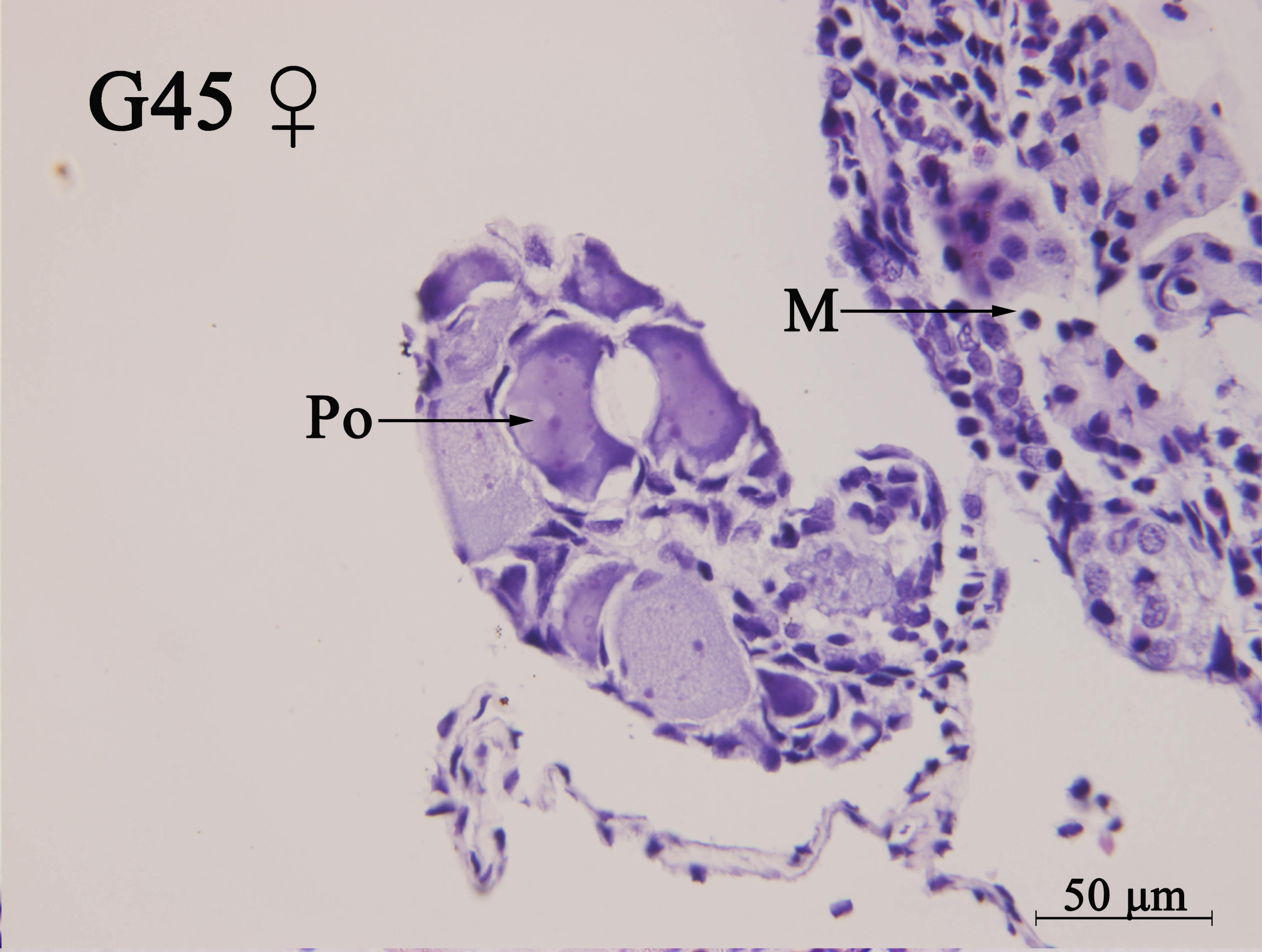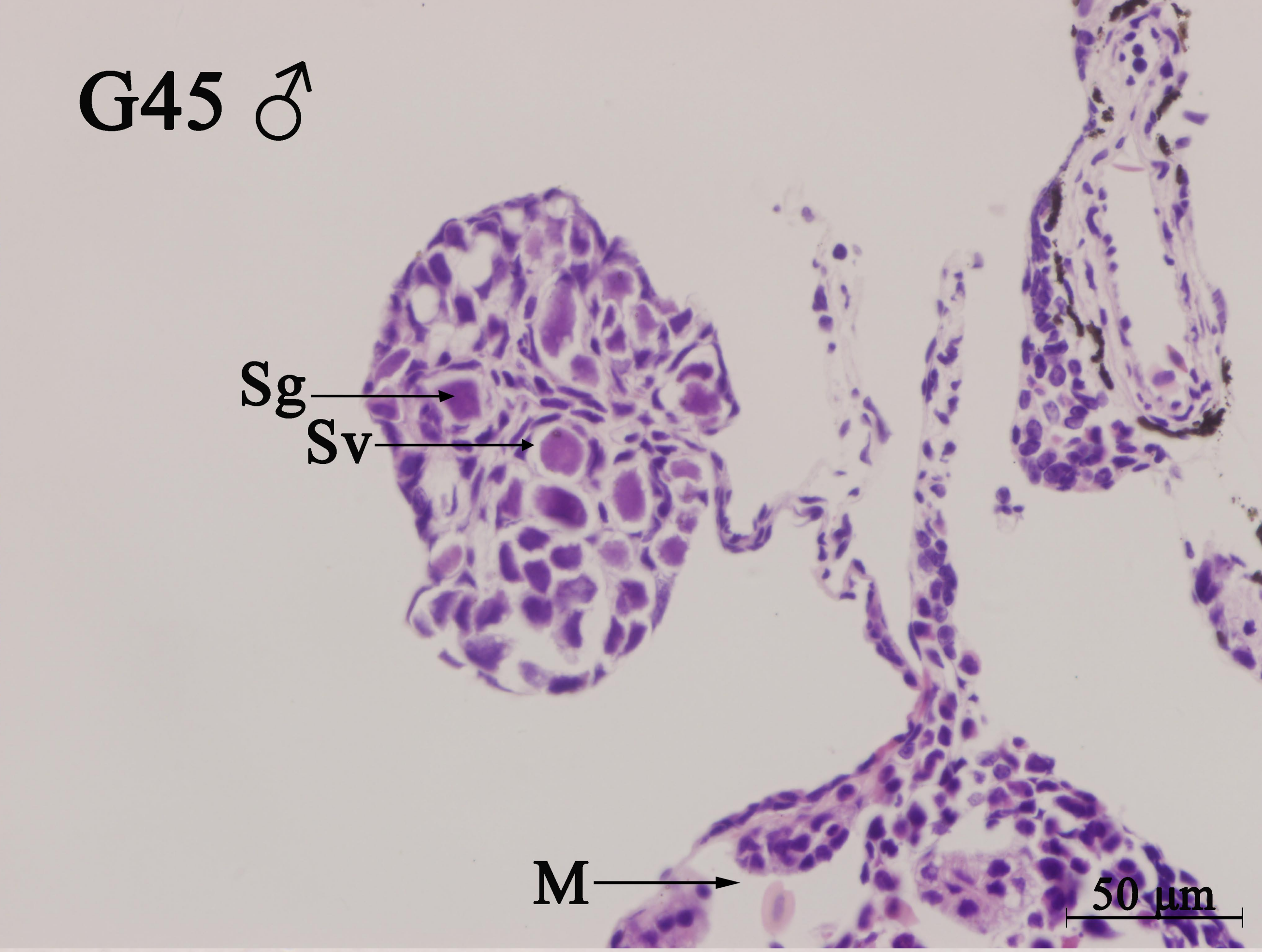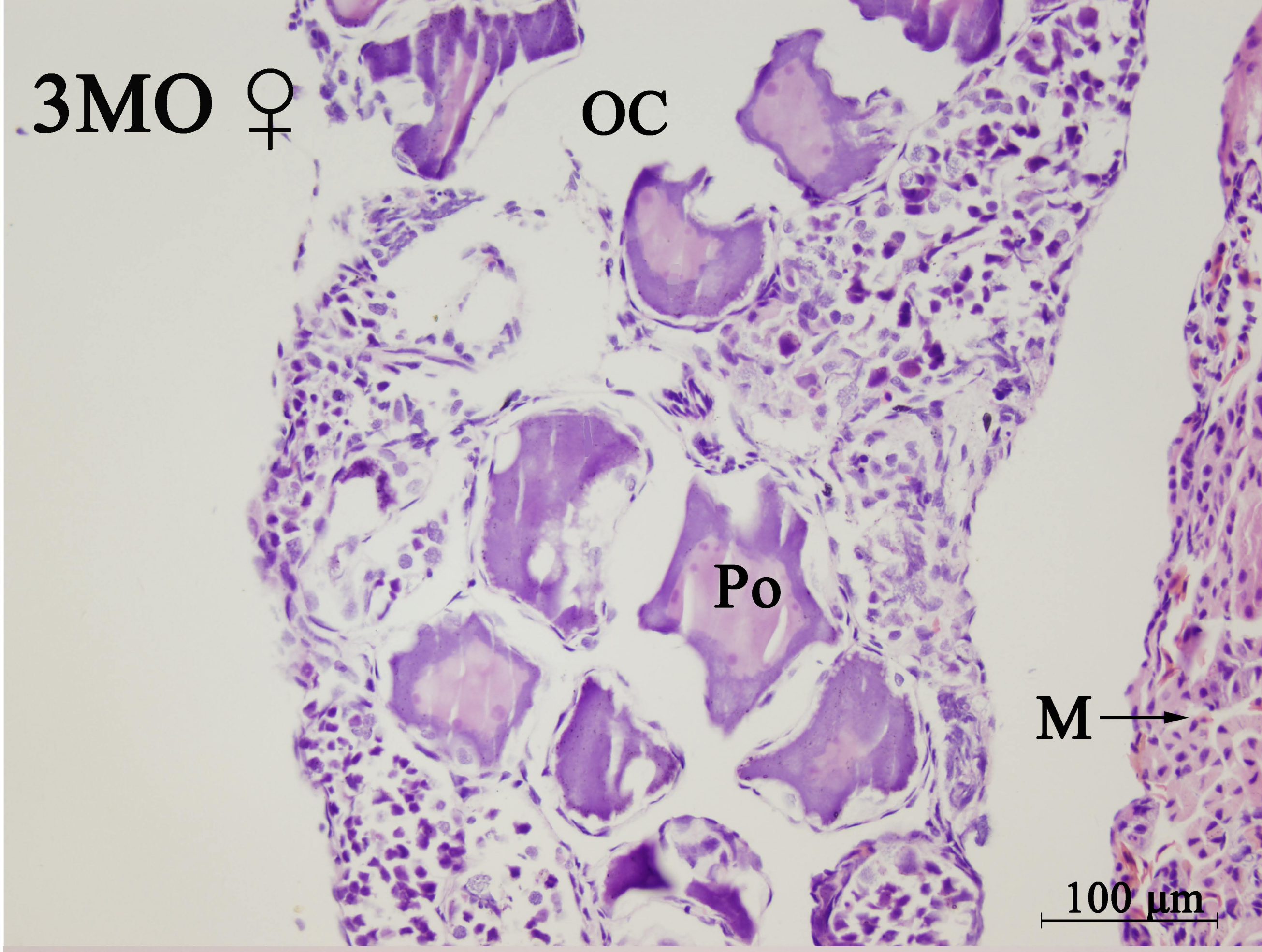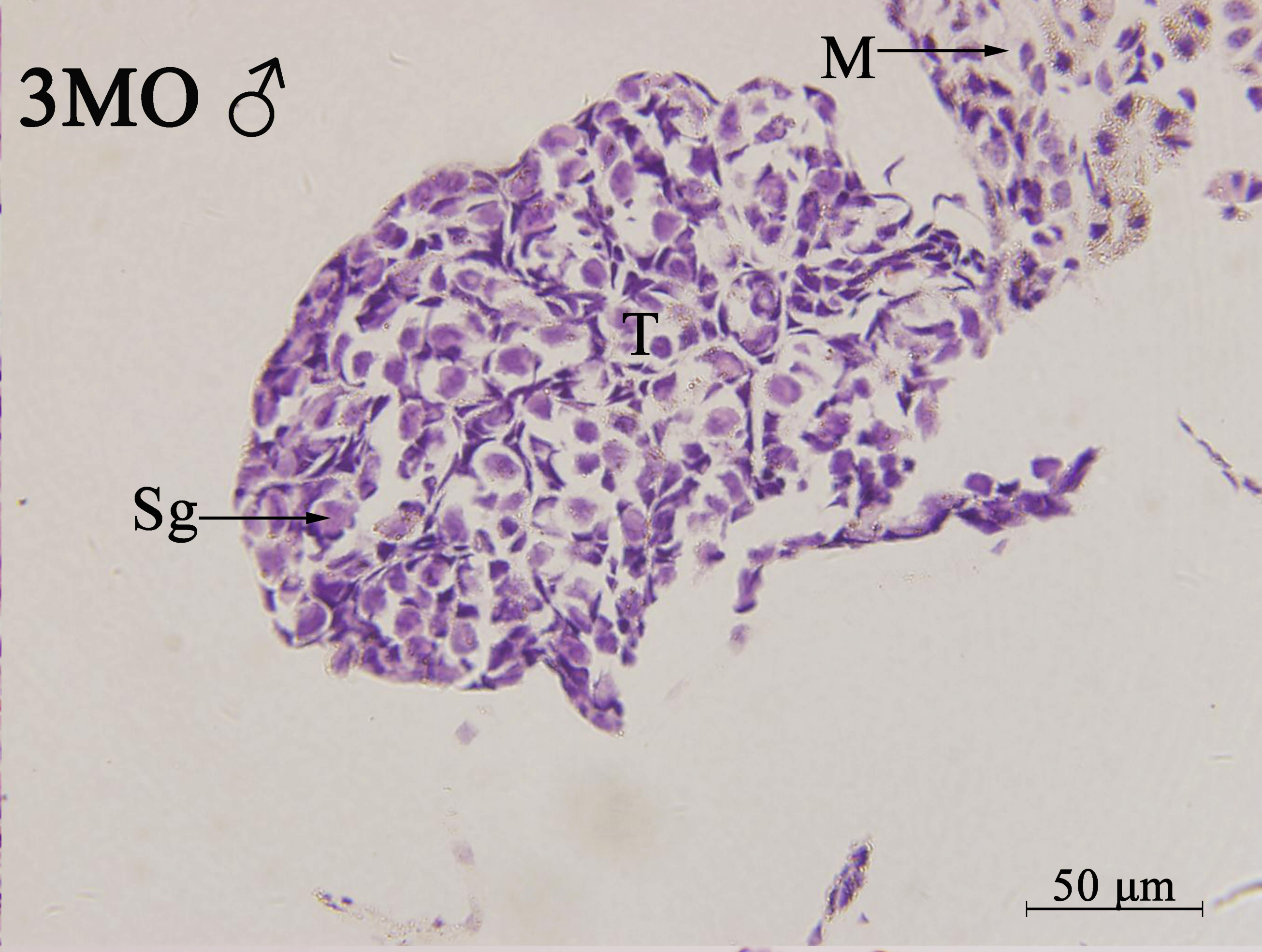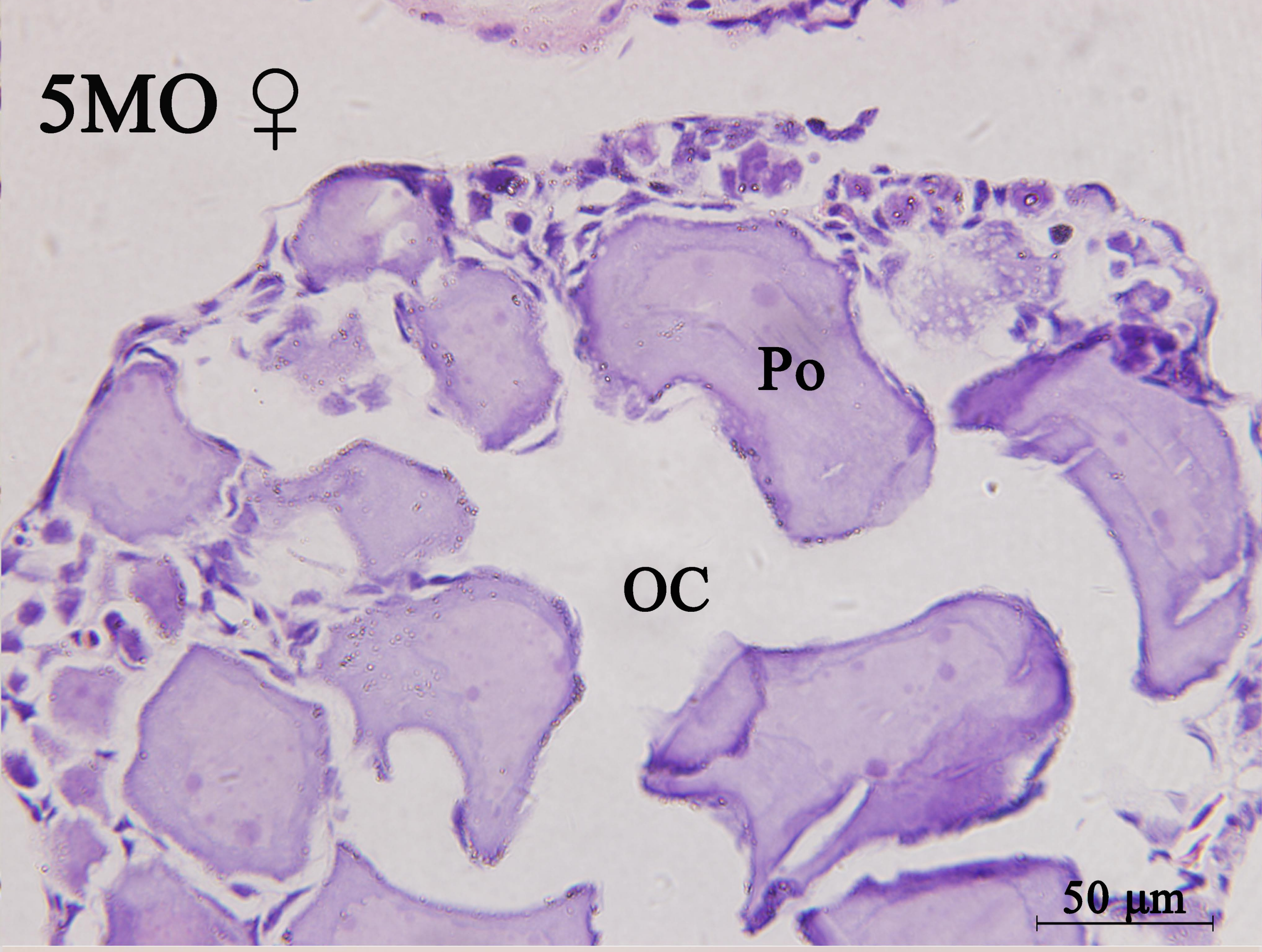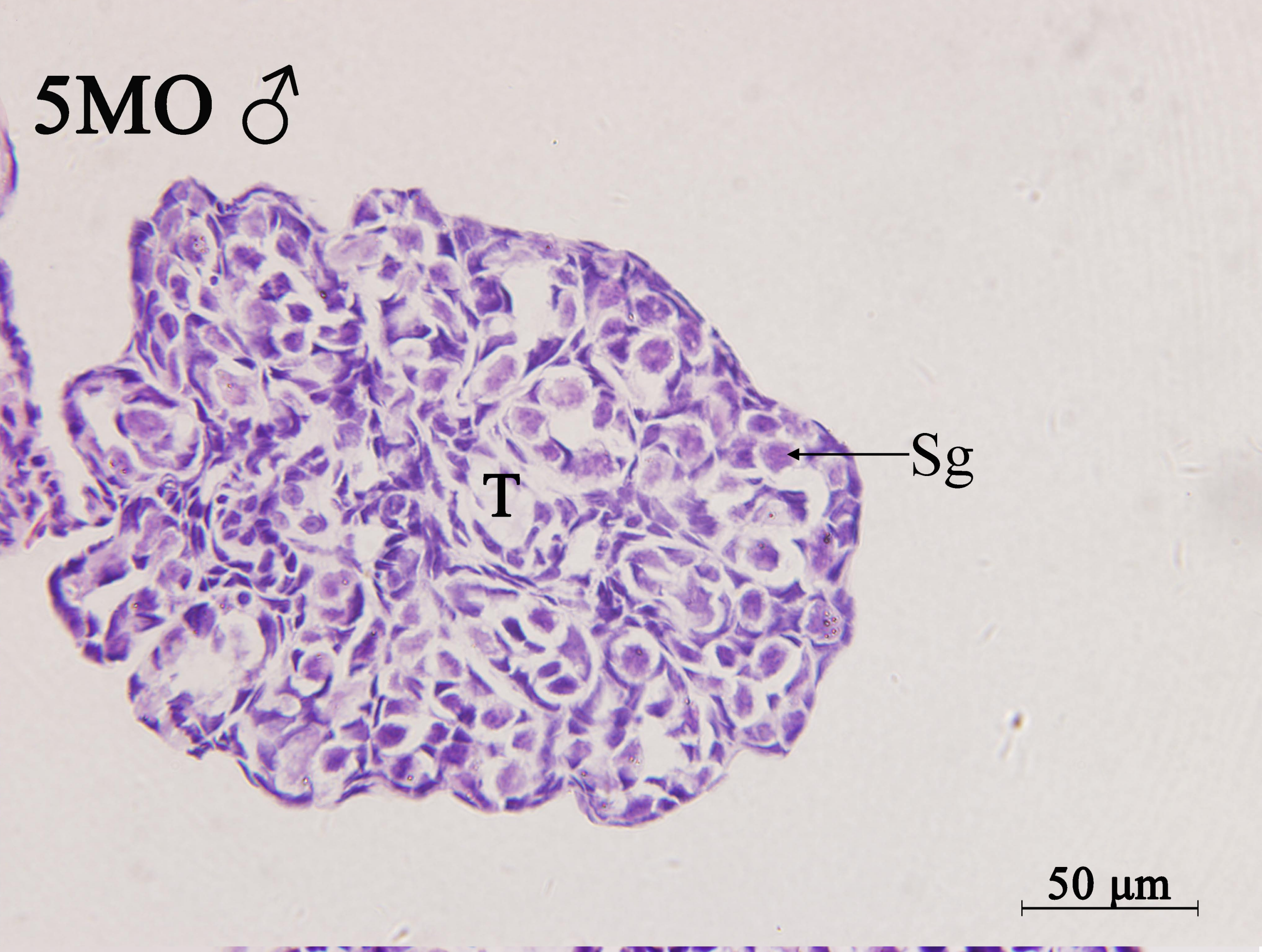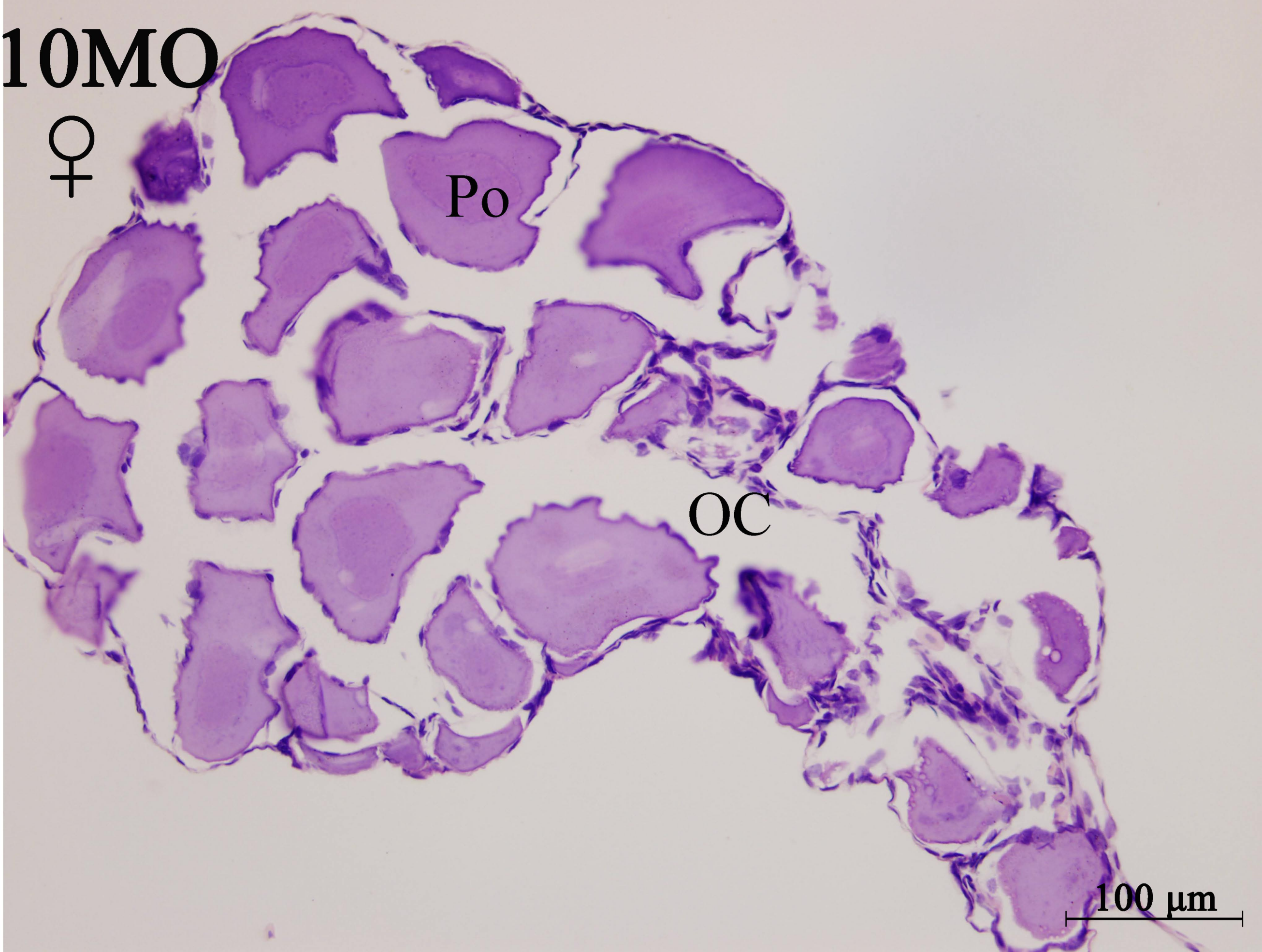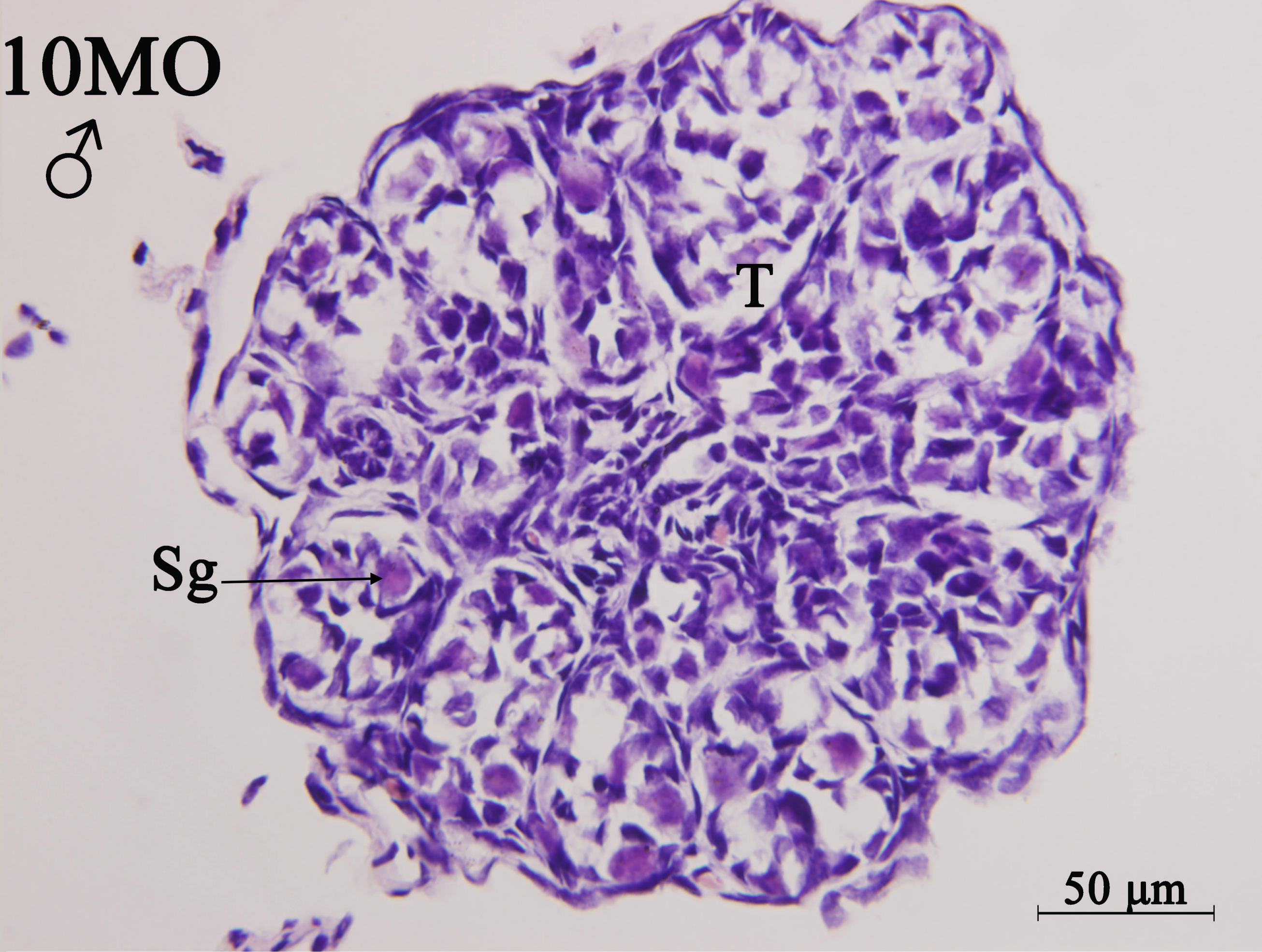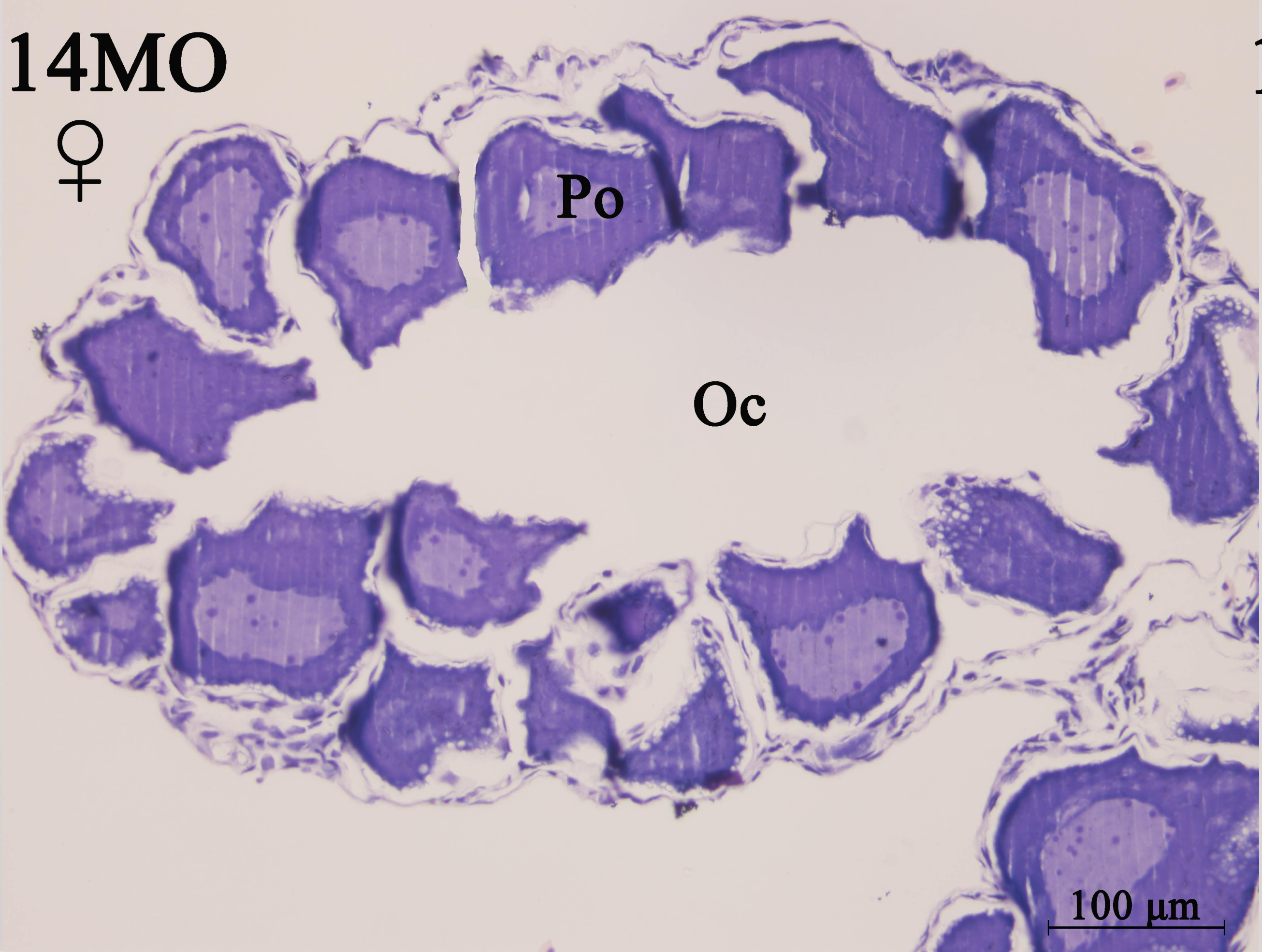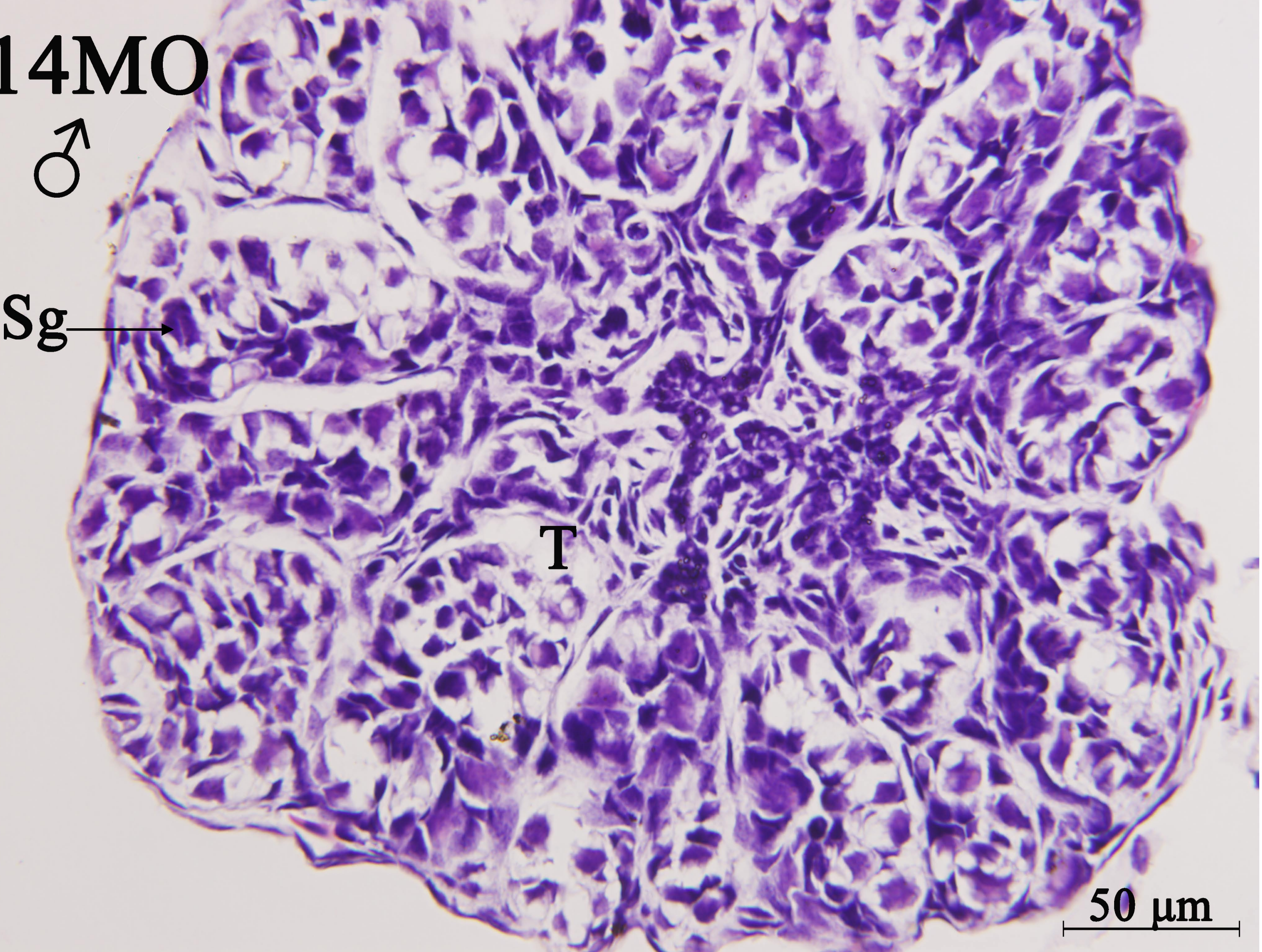

Supplement: Supplementary file 3 — Additional file 3: Fig. S3. Hematoxylin-stained cross-sections of the gonads. Note: M, mesonephros; PGC, primordial germ cell; T, testis; O, ovary; Po, primary oocyte; Oc, Ovarian cavity; Sg, Spermatogonia; S, somatic cell; Sv, seminal vesicles; Pc, Primary cavity; Sc, Secondary cavity; Oo, oogonium; MO, month-old; ♀, female; ♂, male [file 12864_2021_7677_MOESM3_ESM.pdf]

**A**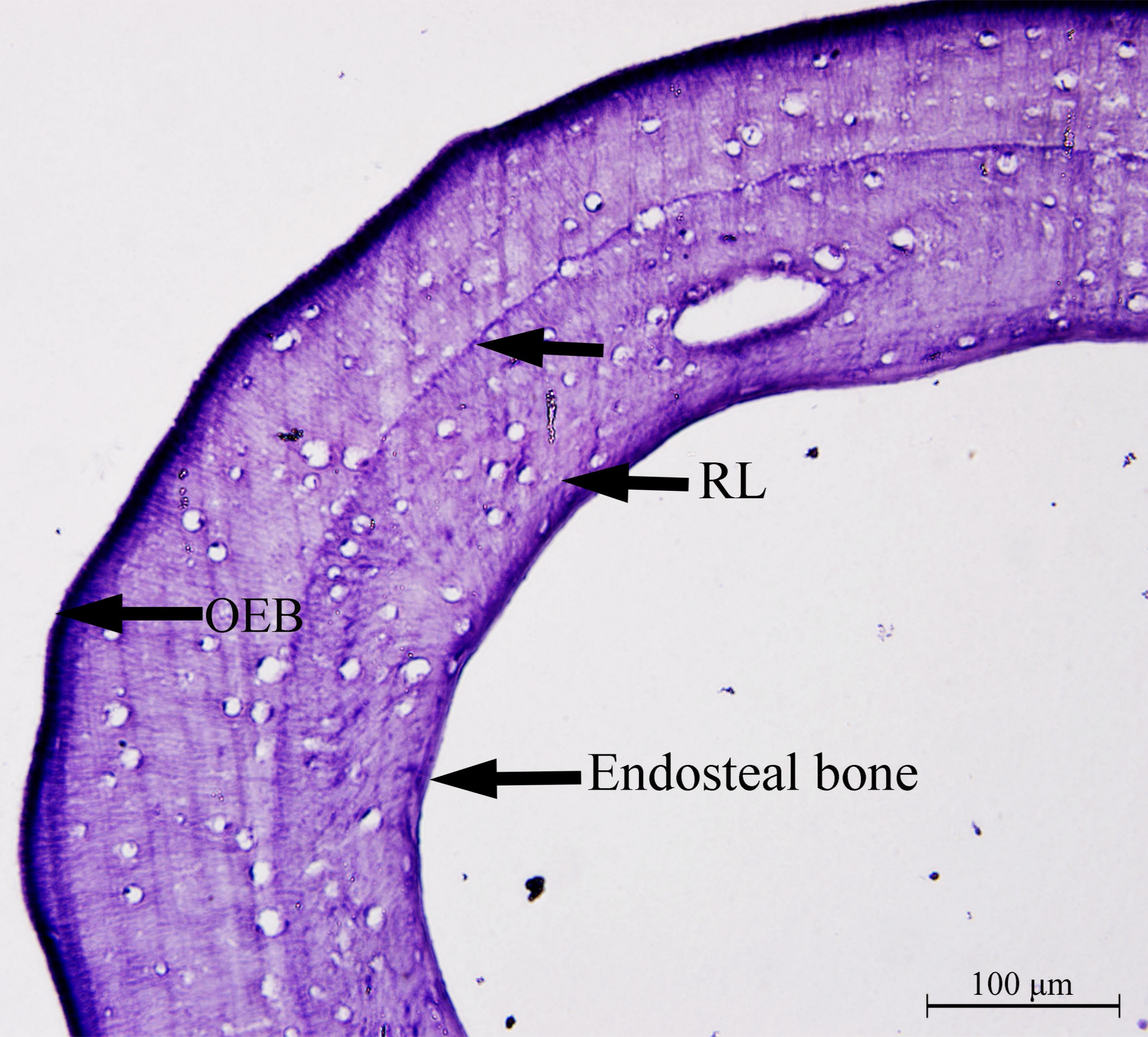**B**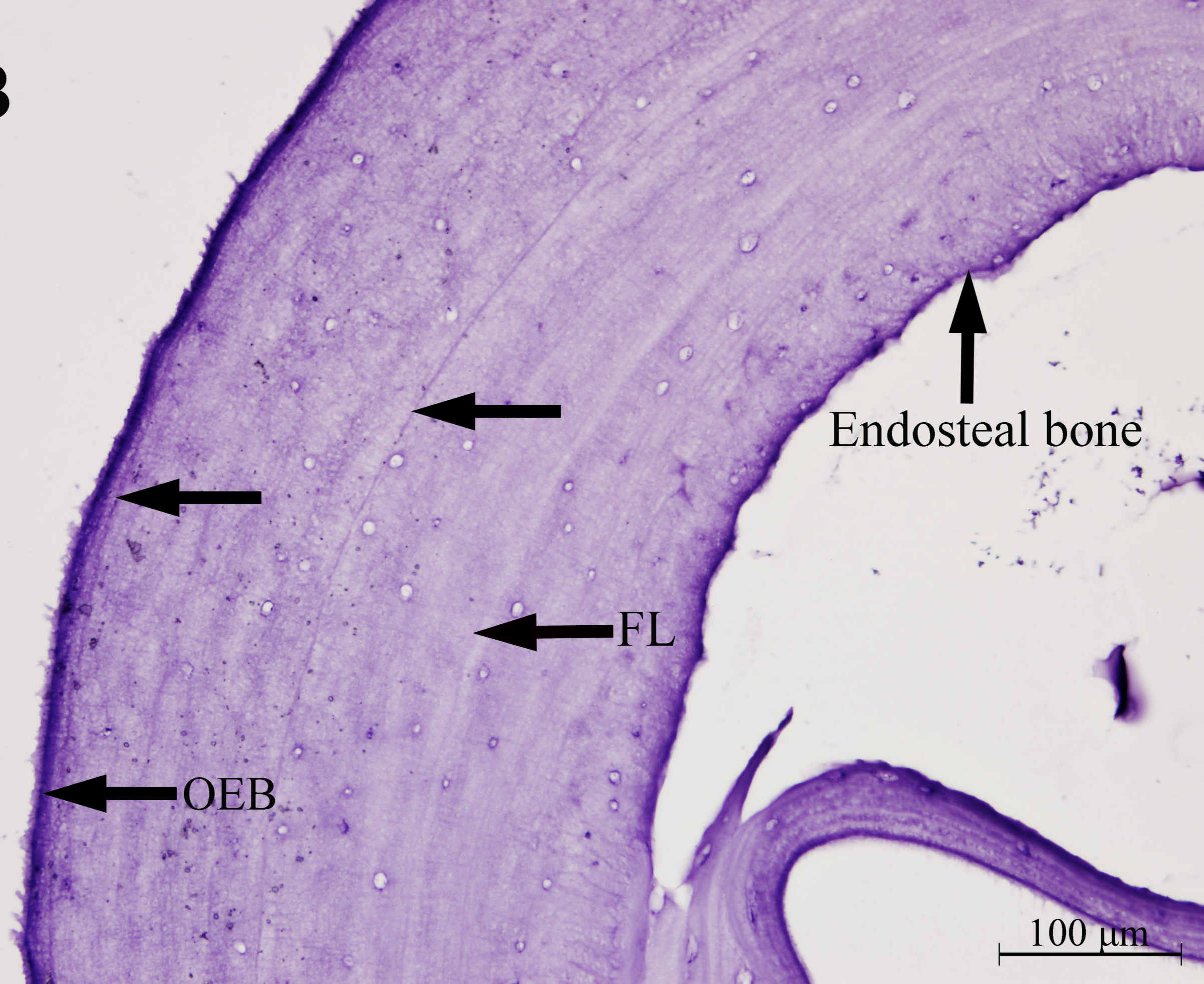**C**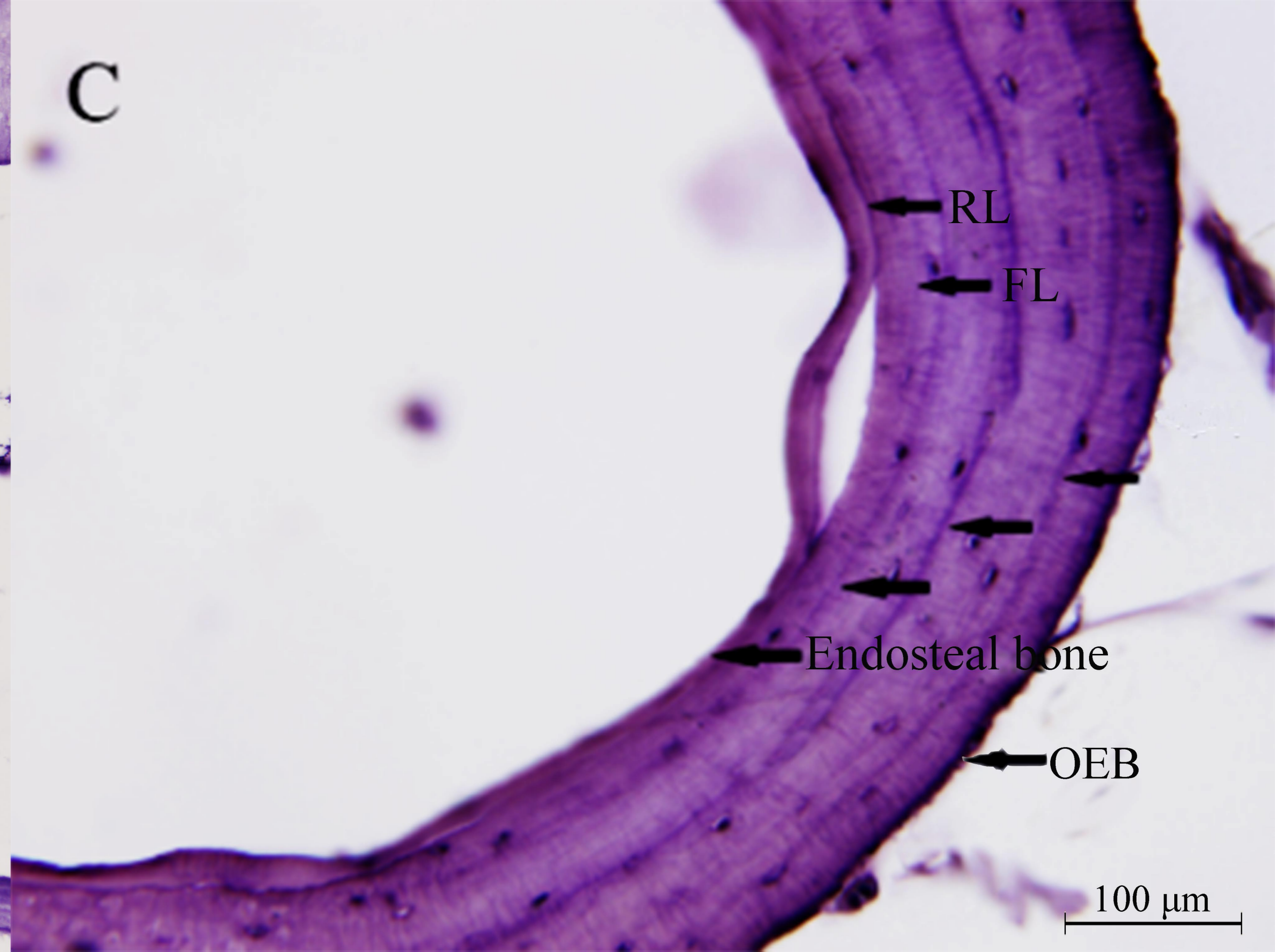

Supplement: Supplementary file 4 — Additional file 4: Fig. S4. Hematoxylin-stained cross-sections of the thigh-bone. The black solid arrow refers to the stagnant growth line. Outer edge of bone (OEB). A, 2-year-old; B, 3-year-old; C, 4-year-old. RL and FL represent resorption lines (the division line between endosteal and periosteal zones) and false line, respectively. Magnification, 200x (bar = 100 μm) [file 12864_2021_7677_MOESM4_ESM.pdf]
